# Suprathreshold perceptual decisions constrain models of confidence

**DOI:** 10.1371/journal.pcbi.1010318

**Published:** 2022-07-27

**Authors:** Shannon M. Locke, Michael S. Landy, Pascal Mamassian

**Affiliations:** 1 Laboratoire des Systèmes Perceptifs, Département d’Études Cognitives, École Normale Supérieure, PSL University, CNRS, Paris, France; 2 Department of Psychology, New York University, New York, New York, United States of America; 3 Center for Neural Science, New York University, New York, New York, United States of America; Scuola Internazionale Superiore di Studi Avanzati, ITALY

## Abstract

Perceptual confidence is an important internal signal about the certainty of our decisions and there is a substantial debate on how it is computed. We highlight three confidence metric types from the literature: observers either use 1) the full probability distribution to compute probability correct (Probability metrics), 2) point estimates from the perceptual decision process to estimate uncertainty (Evidence-Strength metrics), or 3) heuristic confidence from stimulus-based cues to uncertainty (Heuristic metrics). These metrics are rarely tested against one another, so we examined models of all three types on a suprathreshold spatial discrimination task. Observers were shown a cloud of dots sampled from a dot generating distribution and judged if the mean of the distribution was left or right of centre. In addition to varying the horizontal position of the mean, there were two sensory uncertainty manipulations: the number of dots sampled and the spread of the generating distribution. After every two perceptual decisions, observers made a confidence forced-choice judgement whether they were more confident in the first or second decision. Model results showed that the majority of observers were best-fit by either: 1) the Heuristic model, which used dot cloud position, spread, and number of dots as cues; or 2) an Evidence-Strength model, which computed the distance between the sensory measurement and discrimination criterion, scaled according to sensory uncertainty. An accidental repetition of some sessions also allowed for the measurement of confidence agreement for identical pairs of stimuli. This N-pass analysis revealed that human observers were more consistent than their best-fitting model would predict, indicating there are still aspects of confidence that are not captured by our modelling. As such, we propose confidence agreement as a useful technique for computational studies of confidence. Taken together, these findings highlight the idiosyncratic nature of confidence computations for complex decision contexts and the need to consider different potential metrics and transformations in the confidence computation.

## Author summary

The feeling of confidence in what we perceive can influence our future behaviour and learning. Understanding how the brain computes confidence is an important goal of researchers. As such, researchers have identified a host of potential models. Yet, rarely are a wide range of models tested against each other to find those that best predict choice behaviour. Our study had human participants compare their confidence for pairs of easy perceptual decisions, reporting if they had higher confidence in the first or second decision. We tested twelve models, covering all three types of models proposed in previous studies, finding strong support for two models. The winning Heuristic model combines all three factors affecting choice uncertainty with an idiosyncratic weighting to compute confidence. The other winning model uses a transformation where the strength of the sensory signal is scaled according to sensory uncertainty. We also assessed the agreement of confidence reports in identical decision scenarios. Humans had higher agreement than almost all model predictions. We propose using confidence agreement intentionally as a second performance benchmark of model fit.

## Introduction

Perceptual confidence is a metacognitive judgement accompanying a perceptual judgement that is thought to reflect the observer’s belief about the quality or correctness of their perceptual decision. For example, a person is likely to have more confidence in their judgement of whether the road ahead bends left or right on a bright sunny day than on a foggy evening because the chances of making a mistake are higher in the latter scenario. Confidence is a decision about a decision, and so it is often referred to as a *Type 2* judgement to contrast it with the *Type 1* perceptual judgement [1]. Confidence judgements are ubiquitous in everyday life and are one of the many metacognitive evaluations that guide behaviour and learning [2–4].

Numerous models of perceptual confidence have been proposed by researchers, often to capture distinct aspects of decision-making behaviour. For example, some models focus on the relationship between the speed of the perceptual decision and confidence [5] and others on the degree to which confidence reports can distinguish correct from incorrect decisions [6]. The majority of confidence models are process models that extract the confidence decision variable from either the original Type 1 decision process, or a comparable decision process, such as a partially or completely independent reconstruction [7] or further evolved Type 1 decision process [8]. The output of these confidence models is a confidence decision variable that can then be mapped to a behaviour (e.g., pressing a button on a keyboard). Confidence decision variables used by researchers in their modelling can be categorised into three main metric types (Table 1): Probability, Evidence-Strength, and Heuristic.

**Table 1:** Some implementations of the three most common confidence metric types used for modelling perceptual confidence and example study that used this model.

Probability	Evidence-Strength	Heuristic
**Bayesian confidence**: posterior probability of being correct [11] **Log-Probability-Ratio**: of the posterior probability or likelihood [11] **Two-best**: posterior probability difference of the top two choice alternatives [12] **Entropy**: the uncertainty across the posterior distribution of all choice alternatives [12]	**Extended SDT**: distance between measurement & criterion [6, 13] **Drift Diffusion**: diffusion-process state & elapsed time w./w.o. additional accumulation [8] **Balance-of-Evidence**: relative final state of separate accumulators [16] **Supporting-evidence**: strength of decision-consistent evidence only [18, 19]	**Stimulus Variability**: over- or under-weighting of external noise as a cue [14] **Reaction-time**: between stimulus onset and response [15] Other stimulus cues to task difficulty [17]

*Probability* confidence metrics are consistent with a common definition that confidence is the probability that the perceptual decision is correct [9]. This is also referred to as statistical confidence, or Bayesian confidence, if it is computed from a Bayesian posterior probability [10–12]. To compute a probability metric, the observer must consider the probability of all the possible states of the stimulus consistent with their perceptual choice. For example, if they reported a motion direction as clockwise of vertical, this would be the probability of the stimulus being anywhere between 0 − 180° clockwise of the decision boundary. Thus, computation of a full probability distribution is necessary for this confidence decision variable. Bayesian Probability-metric models are typically used as an upper benchmark of confidence performance, as they consider all available information to assess the probability of being correct for reporting their confidence.

In contrast, *Evidence-Strength* confidence metrics are derived from point estimates in the Type 1 decision process and so do not require the representation of full probability distributions. One common Evidence-Strength metric is the *Distance-From-Criterion* (DFC) metric typically used in extended Signal Detection Theory (SDT) models of confidence [13, 20]. In the extended SDT framework, confidence is monotonically related to the unsigned distance between the sensory measurement and the decision criterion. In the case of categorical confidence judgements (e.g., binary report, scale, etc.), additional confidence criteria delineate the mapping between distance and confidence [6, 7, 21]. Most DFC-metric models also allow this sensory measurement to differ from that used for the perceptual decision [22], either by additional confidence noise corrupting the measurement [23–25] and/or from altering the measurement with parallel decision processes [7, 26]. Variants of the DFC metric have been proposed to allow a point estimate of sensory uncertainty to remap the confidence criteria [11, 27, 28], or allow biases in the distance measures to reflect consideration of only choice-congruent evidence [19].

Another common Evidence-Strength metric is the *Accumulator* metric. In accumulation-to-bound models, the Type 1 decision process is represented with one or more decision variables that are updated according to incoming sensory evidence [29]. In this manner, accumulation-to-bound models capture both the final choice and the temporal dynamics of that choice (e.g., reaction time). Thus, both the distance of the Type 1 decision variable from the initial decision state and total decision time are factored into the computation of the confidence decision variable. Often a mapping that corresponds to the probability of being correct is selected by experimenters [5, 30]. That is, the observer uses a point estimate of the decision process and a well-calibrated mapping function to extract the same confidence decision variable they would have got if they computed it from a full probability distribution. Other confidence decision variables of this type consider the relative states of two or more separate accumulators at the time of the decision [16] or partially interacting accumulators [18]. Extended versions of the accumulation-to-bound models allow the final state of the decision variable for the perceptual judgement and the confidence judgement to differ based on additional evidence accumulation between the Type 1 and Type 2 reports [8, 31].

The third metric type is the *Heuristic* metric, an ever-expanding category of confidence decision variables that have been created to capture behaviour beyond that predicted by the favoured standard models mentioned above. While in some cases, the heuristic label has been applied to variants of the Evidence-Strength metric [12, 19, 32], the majority of “heuristic” confidence models have focused on the use of stimulus cues to infer decision uncertainty. Support for this method of computing confidence comes from a series of studies demonstrating that observers over- or under-weight external noise in the stimulus when reporting confidence [14, 33–35]. That is, they display a dissociation between Type 1 and Type 2 performance such that, if perceptual performance is matched for two stimuli with different levels of external noise, confidence is not equated. Other identified heuristic cues are reaction time [15] and task-difficulty variables [17]. Importantly, Heuristic metrics do not require access to the Type 1 decision process, and reflect learned associations between stimulus features or difficulty cues and confidence, which may or may not be well calibrated to reality. However, we note that for all the evidence of heuristic cue use, participants do not always rely on stimulus-uncertainty cues when they are available [36, 37].

These variations in the confidence computation alter how stimulus strength and sensory uncertainty influence confidence. To take an example from our task, consider observers trying to infer the mean of an invisible generating distribution from some generated dot samples, to judge if the mean is left or right of centre. Here, the position of the mean is the stimulus strength, and the sensory uncertainty is inversely related to the number of dot samples drawn. To understand the effect of sensory uncertainty on the confidence decision variable, we will consider two dot-cloud stimuli, one with 2 dots generated from a mean on the left (i.e., high sensory uncertainty) and the other with 3 dots generated from a mean on the right (i.e., low sensory uncertainty). In both cases, the means are equidistant from the centre and the observer correctly identifies the laterality of the generating distribution from the presented dot cloud. But how does their sense of confidence compare in these two scenarios?

**Fig 1:**
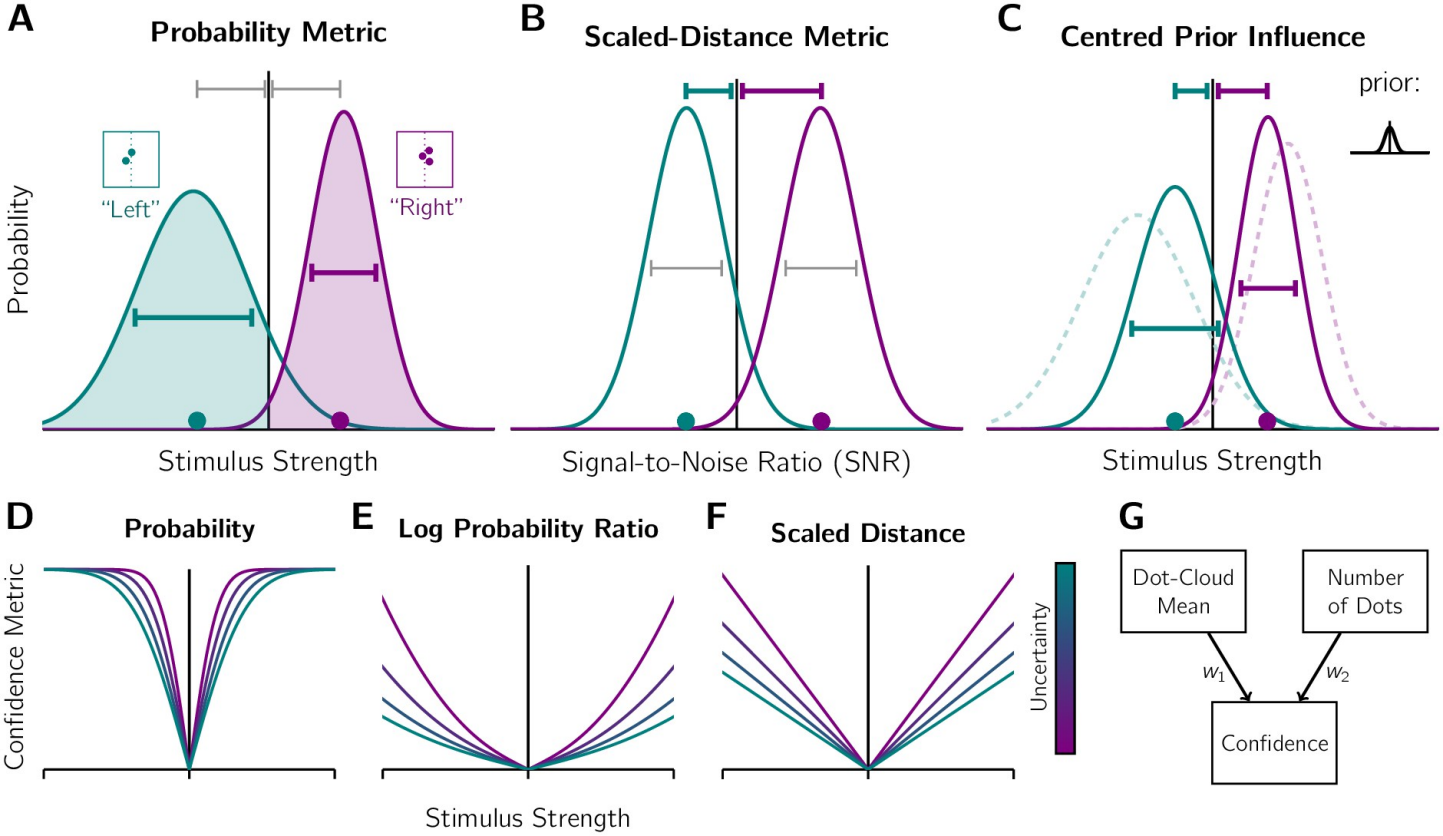
Sensory uncertainty and stimulus strength in the confidence computation. A-C) Extracting the confidence decision variable from the Type 1 decision process. Scenarios contrasted: dot cloud with 2 dot samples (teal; high uncertainty; see inset for stimulus) and a 3-sample dot cloud (purple; low uncertainty), with both dot-clouds’ means equidistant from the screen centre (dotted line of inset). The observer correctly identifies the lateralisation of the generating distribution’s mean, as shown by their response in quotation marks. Vertical line: decision boundary. Horizontal lines: similarities (grey) or differences (coloured) between the distributions. A) Probability metric. Curves: normalised likelihood functions of the distribution mean, given the sensory measurement (marker; the most likely measurement selected for illustrative purposes). Shaded region: probability of the judgement being correct. The shaded region is greater for the 3-dot scenario, so the observer has higher confidence in this judgement. B) Scaled-distance metric. A signal-to-noise ratio transformation is applied to the distributions in A (i.e., rescaled to units of standard deviation while the areas under the curve on either side of the Type 1 criterion are preserved). The rescaled sensory measurement in the 3-dot scenario has a greater Distance-from-Criterion (DFC) and is judged as more confident. C) Influence of a centred prior (see inset). The posterior distributions (continuous curves), computed according to Bayes’ Rule, are differentially shifted towards the centre from the likelihood function locations (dashed). The 2-dot scenario is shifted more because of its higher uncertainty. Consequently, Probability metrics and DFC metrics yield higher confidence for the 3-dot scenario. D-F) How the confidence metric is affected by stimulus strength and sensory uncertainty (low to high uncertainty represented by colours ranging from purple to teal). The greater the stimulus strength (i.e., distance of mean from centre), the larger the confidence metric. However, raw probability values asymptote at 100% confident (D), whereas the confidence decision variable is unbounded if a Log-Probability-Ratio transformation is applied to the probability of being correct (E) or an Unscaled- or Scaled-Distance metric is used (F). Sensory uncertainty affects the rate of change of the confidence metric in response to changes in stimulus strength. Note *y*-axes have been rescaled for illustrative purposes. G) Heuristic confidence metric computed from estimates of stimulus strength (dot-cloud mean) and sensory uncertainty (number of dots) without consideration of the Type 1 process. The observer sets the weights on these factors, *w*_1_ and *w*_2_.

Fig 1A depicts how an observer using a probability metric would assign higher confidence in the 3-dot scenario, as the probability of being correct (i.e., the shaded region) is greater due to the difference in spread (i.e., sensory uncertainty). Note that the two probability distributions are centred on the two sensory measurements, which, in this case, are the most likely sensory measurements and so are equidistant from the unbiased decision criterion (i.e., are matched in terms of stimulus strength). The behaviour of the Probability metric for different stimulus strengths also depends on whether any transformations are applied. As shown in Fig 1D, the probability metric approaches a ceiling of 100% probability of being correct if stimulus strength is increased. Even with higher sensory uncertainty, this ceiling is approached relatively quickly with slightly greater stimulus strengths. However, the probability of being correct for a binary Type 1 decision could also be expressed as a Log-Probability Ratio (LPR), which is the log of the ratio of the probability of being correct for the selected Type 1 choice to the probability of being correct if you had selected the other Type 1 option [11, 13, 38]. In this scenario, the confidence metric is unbounded, and the difference in the metric between low and high uncertainty increases for larger stimulus strengths (see Fig 1E). So while the relationship of greater confidence for lower uncertainty is typically preserved, the underlying confidence decision variables can differ dramatically depending on transformations in the computation.

An observer who uses the standard DFC Evidence-Strength metric would not behave the same as an observer using the Probability metric. As both measurements have the same DFC, the observer would have the same degree of confidence for the 3-dot and 2-dot scenarios. It is only if the observer scales this measurement according to the degree of uncertainty (i.e., computes the signal-to-noise ratio (SNR)) using a point-estimate of sensory uncertainty, would confidence be greater for the 3-dot scenario (Fig 1B). The effect of this scaling means the sensory measurement is now further from the decision boundary for the the 3-dot scenario compared to the 2-dot scenario. A similar reasoning would apply for an accumulation-to-bound Evidence-Strength metric, where the sensory measurement (i.e., final decision state) and decision time (proportional to uncertainty) jointly determine the degree of confidence. The value of a DFC Evidence-Strength metric scales linearly with stimulus strength. In the case of a SNR scaling, the sensory uncertainty affects the slope (Fig 1F).

Fig 1C depicts yet another way sensory uncertainty can influence confidence. If the prior expectation about the stimulus is concentrated at the decision boundary (e.g., the generating mean is likely to be at or near the screen centre), the current observation can be biased towards this expected stimulus. This enhances the difference between the 3-dot and 2-dot scenarios for both a Probability metric and a DFC Evidence-Strength metric. In fact, even an unscaled DFC Evidence-Strength metric would now reflect greater confidence for the 3-dot scenario due to the prior biasing effect being stronger for measurement from the 2-dot scenario. Note that we are defining different states of the Type 1 decision process in Fig 1A–1C, with the metric type reflecting different degrees of access or use of the decision-process information by the metacognitive system (i.e., full probability distributions or only point estimates). Thus, there is no conflict when an observer, who uses the prior for their Type 1 decision, only uses a point estimate of a biased sensory measurement to compute an unscaled DFC metric for confidence.

Finally, an observer computing a heuristic confidence metric considers the number of dots as well as mean position of all dots as independent inputs without considering an underlying Type 1 process. They then combine these inputs with some weighting scheme of their choice (Fig 1G). If they correctly apply the rule of lower confidence for fewer dots, even if it imperfectly captures the relationship between number of dots and external noise, there will be an effect of sensory uncertainty on confidence.

Despite the diversity of potential confidence models, only a few studies have directly compared models of different metric types. One approach has been to compare the behavioural signatures of competing models. Studies using this approach have tended to compare a Probability metric with a Heuristic metric, finding support for the probability metric [10, 36] or differing support for each metric depending on the perceptual task [35]. Formal model comparisons, on the other hand, have been focused on Probability versus Evidence-Strength metrics with mixed results (note that this is according to our metric definitions not those of the authors who used the term “heuristic” more liberally). Aitchison et al. [32] investigated simultaneous versus sequential Type 1 and Type 2 reports, finding the Bayesian-confidence probability metric best fits sequential reports, but this metric and an Evidence-Strength metric could explain simultaneous reports equally well. Adler & Ma [11] investigated categorisation behaviour when category means differed but variances were matched versus when category variances differed but means were matched. They found that an Evidence-Strength metric with quadratic sensory-uncertainty-dependent bounds best fits the behaviour in both tasks. Lisi et al. [39] tested if confidence could be used in a subsequent decision where the perceptual evidence depended on the correctness of previous perceptual choice, and found that the model with a discrete Evidence-Strength metric outperformed the Bayesian Probability-metric model. Finally, Li & Ma [12] investigated various Probability and Evidence-Strength metrics in the context of a three-alternative forced-choice task. They found a Probability metric that compares the posterior probabilities of the two best alternatives outperformed other metrics. Thus, even with direct comparison of the models, the evidence is mixed for the different metric types and further work is needed to understand the nature of the confidence decision variable. This need was highlighted recently by visual metacognition researchers who stated that determining how confidence is computed with detailed and falsifiable models is important for the field [40].

**Fig 2:**
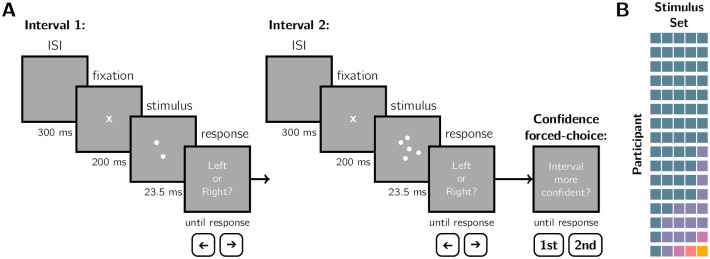
Experimental methods. A) Task design. Observers were shown dots drawn from a Gaussian generating distribution with seven possible horizontal spatial offsets between ±4° from the screen centre. They judged if the distribution mean was left or right of centre. After each pair of perceptual decisions (Interval 1 and Interval 2), they reported the interval in which they had higher confidence in their decision. There were six levels of stimulus uncertainty, defined by information quantity (number of dots: 2 or 5) and quality (sampling distribution SD: 1.5, 2, or 2.5°), presented in an interleaved design. B) Occurrence of unique stimulus sets (colour coded). Squares: stimulus set in a single session. Sets are ordered by type, not session order, and participants by frequency of stimulus set repeats (5-pass to 1-pass).

The aim of the present study was to compare the fit of confidence models of all three metric types to the behaviour of humans performing a visual decision-making task. Specifically, observers had to infer if the mean of a dot-generating distribution was left or right of centre (Fig 2A). We employed a mixed-difficulty design, varying the position of the mean (i.e., stimulus strength), as well as the spread of the dot-generating distribution (the *quality* manipulation) and the number of dots drawn (the *quantity* manipulation). Both of the quantity and quality manipulations affected sensory uncertainty. This design allowed us to assess how the observer took sensory uncertainty into account in the computation of confidence. We used the confidence forced-choice method [26, 41]: after two consecutive perceptual decisions, the observer reports whether they had greater confidence in the first or second decision. This allowed us to investigate suprathreshold perceptual decisions where confidence is typically high without being concerned with ceiling effects (e.g., always reporting high confidence or the highest scale rating). We targeted this difficulty regime because the confidence metrics are particularly divergent for more extreme stimulus strengths (Fig 1D–1F). We selected seven base models (twelve models total considering variants in the prior distribution) that captured the diversity of potential confidence computations illustrated in Fig 1 (models described in Table 2). In addition to formal model comparison, we also investigated a new, qualitative, behavioural signature of confidence: *confidence agreement*. Due to a coding error, many sessions were identical repeats, in some cases as many as 5 repeats (Fig 2B). We compared the confidence agreement of the observers to the confidence agreement of model simulations using the best-fitting parameter values as a benchmark of model fit.

**Table 2:** Summary of the seven base models. Models spanned all three metric types and considered different implementations of confidence noise, prior distributions, and confidence-variable transformations. Standard unbiased Gaussian confidence noise is used unless noted otherwise. There are twelve distinct models for comparison when including the prior variants.

Model	Type	Description
Ideal Conf. Observer	Probability	Compares the p(correct) for each decision, computed from the posterior distribution. Only the centred-prior variant considered for this model.
Basic Probability	Probability	Compares the p(correct), but with early confidence noise (beta, constrained [0, 1]) applied before the comparison, interval bias, and two prior variants considered (flat & centred).
Probability Difference	Probability	Compares the p(correct), but with late confidence noise applied after the comparison, interval bias, and two prior variants considered (flat & centred).
Log Probability Ratio	Probability	Compares the p(correct), but with a LPR transformation applied, confidence noise, interval bias, and two prior variants considered (flat & centred).
Unscaled Distance	Evidence Strength	Compares the DFC of point-estimates of the Type 1 decision process, with confidence noise and interval bias. Two prior variants considered (flat & centred).
Scaled Distance	Evidence Strength	Compares the DFC of SNR-scaled point-estimates of the Type 1 decision process, with confidence noise and interval bias. Two prior variants considered (flat & centred).
Heuristic	Heuristic	Compares a weighted sum of the estimated difference in stimulus strength and estimate difference in sensory-uncertainty factors, with late confidence noise and interval bias.

To preview the results, we found that the confidence judgements were affected by both the quantity and quality manipulations and all three metric types were supported by at least one observer. The overall best-fitting model at the group level was the Heuristic model, followed closely by a DFC Evidence-Strength model where evidence strength was scaled by the sensory uncertainty. Both of these models best-fit an equal number of participants. Together, our results indicate heterogeneity in confidence strategy; observers were unlikely to compute full probability distributions for confidence and were more likely to rely on stimulus-based heuristic cues or summary statistics of the decision process. For confidence agreement, the best-fitting model, on a per-participant basis, almost always underestimated the observers’ confidence agreement. This finding suggests there is still room for improvement in the modelling of perceptual confidence.

## Results

### Confirming that the stimulus manipulations affected confidence

**Fig 3:**
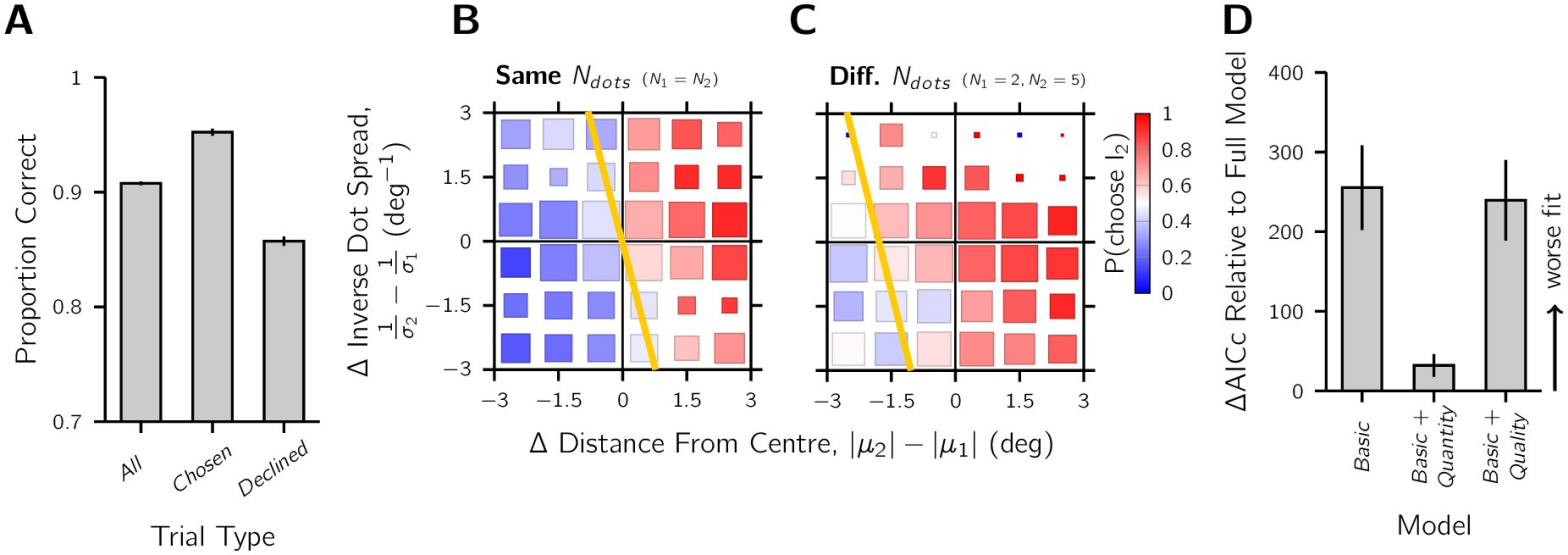
Confidence manipulation checks. A) Suprathreshold Type 1 task performance. The mean proportion of correct spatial judgements across observers is shown for different trial categories. The left-most bar shows performance for all trials, and the next two bars for trials sorted by confidence, either being in the interval chosen as more confident or declined. B-C) Raw confidence choices, sorted by stimulus properties across the two intervals of a confidence pair. The colour code represents the proportion of “Interval 2” as more confident choices averaged across observers. Confidence choices are plotted as a function of the difference in distance from centre across intervals and difference in inverse dot spread. The distance from centre and dot spread were calculated using the empirical mean and SD of the dots displayed, and binned in the range ±3° for plotting. Gold line: the confidence-indifference contour, where the observer is equally likely to report Interval 1 or 2, calculated from the Full model in the nested logistic regression analysis. B) Comparisons where the number of dots was the same in each interval. C) Comparisons where the number of dots differed, with stimulus information and confidence selectively flipped so that Interval 2 has more dots for plotting purposes. D) Model comparison for the nested logistic regression analysis. AICc scores are reported relative to the Full model (winner) that contained both quantity and quality predictors. The Basic + Quantity and Basic + Quality models only contained one of these predictors and the Basic model contained neither. The results show that both the quantity and quality manipulations affected confidence. Larger positive scores indicate a worse fit. Error bars: ±SEM.

First, we examined whether the confidence reports meaningfully distinguished accuracy in the Type 1 spatial task (Fig 3A). Observers had an overall high level of accuracy of 0.91 ± 0.002 (mean ± SEM, *μ*_*cloud*_ = 0° trials excluded from calculation). Despite this near-ceiling performance, the interval chosen as more confident was on average more likely to be correct than the declined interval in the pair: 0.95 ± 0.003 versus 0.85 ± 0.004 (*t*_15_ = 14.96, *p* < 0.01). The low variance in performance across observers was due to two factors: 1) the exclusion of the ambiguous *μ*_*cloud*_ = 0° trials leaving only the easy near-ceiling conditions; and 2) error trials being largely due to the sampled dots favouring the side opposite to the mean and therefore common responses were given across repeated sessions and different observers. The latter factor is evident from the even better performance results if responses are scored according to the mean of the dots displayed on the screen (i.e., the centroid), removing the factor of external noise on performance: 0.94 ± 0.003 for all, 0.98 ± 0.003 for chosen, and 0.90 ± 0.004 for declined (*t*_15_ = 19.55, *p* < 0.01). In sum, these results indicate that observers made meaningful confidence forced-choice judgements.

The effect of the quantity and quality manipulations on confidence can be seen in the raw response data (Fig 3B and 3C). In the plots, positive difference values favour Interval 2 as more confident choices (red) and negative differences Interval 1 choices (blue). For all trials, the further the dot cloud was from the screen centre, compared to the stimulus of the other interval, the more likely it was to be chosen as more confident. When the two intervals had a different number of dots (Fig 3C), the interval that contained the greater quantity of dots was more likely to be chosen as more confident. Note in Fig 3C, the interval with more dots was coded as Interval 2 for plotting purposes. Finally, when the two intervals differed in dot-cloud spread, computed as the horizontal standard deviation in the presented dots, an interval was more likely to be chosen as more confident if it had the smaller spread. Smaller spread corresponds to larger inverse spread (1/*σ*) in Fig 3B and 3C.

To quantitatively confirm both the quantity and quality manipulations affected confidence, we performed a nested logistic regression analysis. The full model, which had the difference in distance from the screen centre, inverse dot spread, and number of dots as predictors, outperformed simpler models without the quantity and/or quality predictors (Fig 3D). See Fig B in S1 Text for more details on the models and model comparison. The gold confidence-indifference lines in Fig 3B provide a visualisation of the fit of the full model.

Finally, we investigated if the set-repetition affected behaviour. For each set, we computed the proportion correct and the difference in proportion correct for chosen versus declined trials to reflect discrimination performance and metacognitive sensitivity respectively. Values were normalised using a participant-specific z-score and then sets grouped by their repeat number. There were fewer sets in higher repeat bins: 28, 16, 15, 13, and 8 sets for 1–5 repetitions respectively. Unbalanced 1-way ANOVA tests revealed a significant effect of repetition number on discrimination behaviour (*F*_4,75_ = 3.41, *p* < 0.05) but not metacognitive sensitivity (*F*_4,75_ = 1.9, *p* > 0.05). A multiple pairwise comparison of group means for normalised percent correct reveal that the discrimination performance in the fifth repetition was significantly lower than the second and third repetitions. Given that fifth repetitions were always the final testing session, we interpret this effect as related to motivation in the task being lower on the final day of testing rather than set-repetition specifically.

Together, these results suggest observers’ confidence computations were affected by the stimulus strength as well as both sources of sensory uncertainty in this easy perceptual task. To better understand the computation of the confidence decision variable, we next examine several process models that capture the full decision process (i.e., both perceptual and confidence judgements).

### Type 1 model comparison results

**Fig 4:**
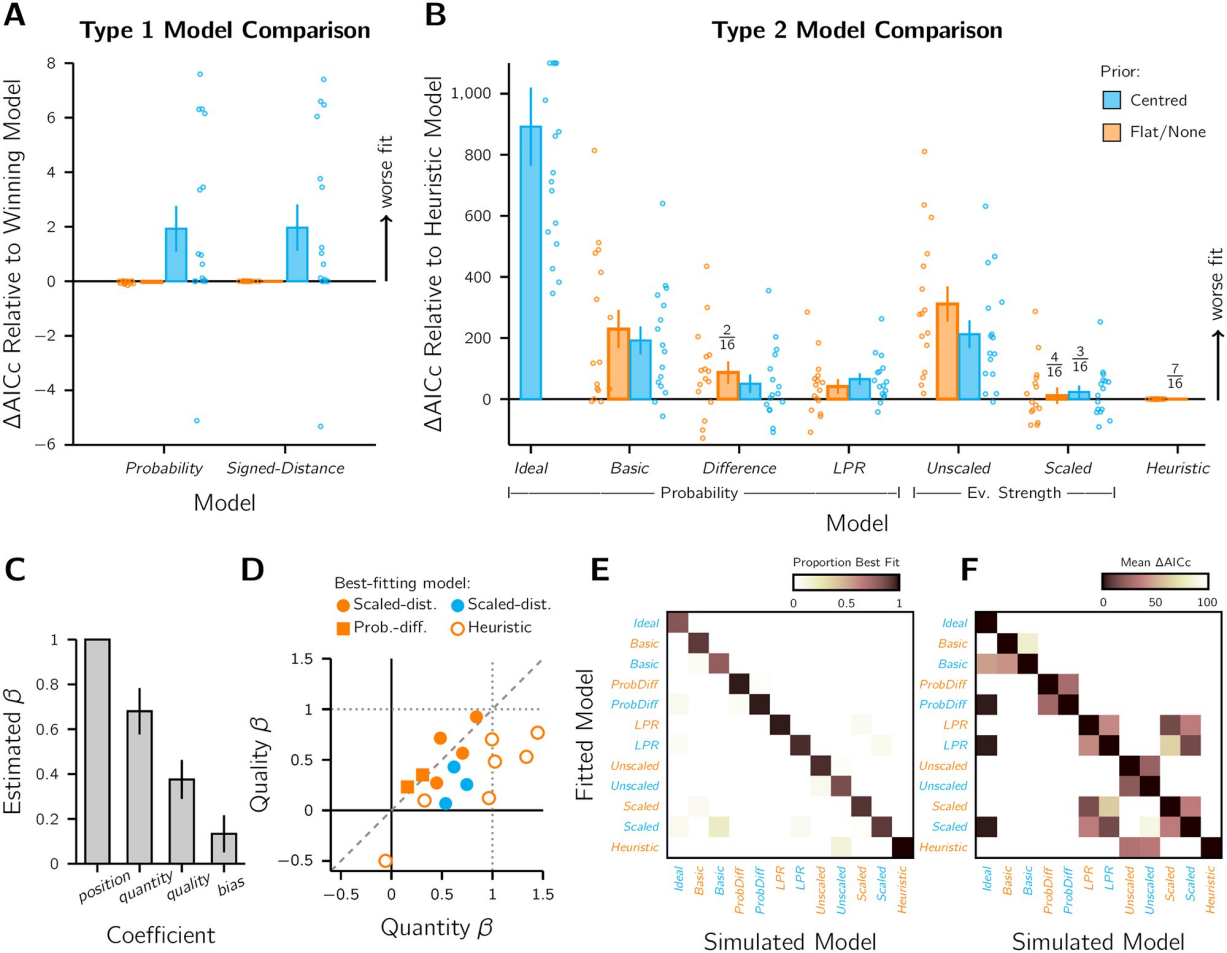
Model fit results (*n* = 16). A) Relative AICc scores for the Type 1 models. Scores were compared to the winning flat-prior variant models. Bars: average relative AICc score. Markers: Individual participant results. Colour: flat-prior (orange) or centred-prior (blue) variant. Error bars: ±SEM. B) Relative AICc scores for the Type 2 models, with fraction best-fit annotated. Models are grouped by metric type. Note the different y-axis scales between panels A and B. C) Average best-fitting Heuristic-model coefficients across all observers. Note that the position coefficient was always fixed at 1 in the model, but is graphed to illustrate the relative coefficient weights. Error bars: ±SEM. D) The best-fitting quantity and quality coefficients per observer. Marker colour and type indicates the best-fitting model for that observer. Dotted lines: the position coefficient value for comparison. Dashed line: equality line. E) Model recovery results. For each model and observer, 10 data sets were simulated using the participant’s best-fitting parameters (1920 data sets total) and then fit by each of the 12 models. The best-fitting model was tallied per dataset per simulated model. Dark squares along the downward diagonal indicate high model recovery success. F) The relative AICc scores compared to the simulated model (downward diagonal of 0).

Four Type 1 models were considered. All models had three free parameters: a perceptual decision criterion (*k*_1_), per-dot sensory noise (*σ*_*dot*_), and a lapse rate (λ). The models differed in two ways: 1) whether the posterior distribution was computed with a flat or centred prior, and 2) if the Type 1 decision variable was the probability that the dot-cloud was on the left/right or the point estimate of the posterior’s mode relative to the decision criterion (both metrics are always in 100% agreement). The two prior-variants are also often in agreement about the perceptual choice, except for cases close to a biased decision boundary. Thus, it was unlikely to see large differences in the Type 1 model-comparison results. As expected, we found identical fits for the probability and signed-distance metrics (Fig 4A), and near-identical fits for the prior variants. The purpose of fitting all four models was to ensure that the Type 1 parameters could be fixed accordingly based on the Type-1 responses for the Type 2 model fits.

### Type 2 model comparison results

Twelve Type 2 models were compared, with confidence computed with a Probability metric in seven models, a DFC Evidence-Strength metric in four models, and a Heuristic metric in one model (Fig 4B). A summary of the models can be found in Table 2, the model equations in Table 3, and the mean and spread of the best-fitting parameter estimates per model in Table A in S1 Text. The Heuristic model had the best AICc score averaged across observers, with 7 of the 16 observers best fit by this model. The Scaled-Distance models were a strong competitor, with 4 observers best fit by the flat-prior variant and 3 by the centred-prior variant. The average AICc scores for these variants were respectively 10.8 ± 26.9 and 23.1 ± 20.7 higher than the Heuristic model. The flat-prior variant of the Probability-Difference model best fit 2 observers and had an average relative AICc score of 41.0 ± 23.8. Overall, the Ideal, Basic-Probability, and Unscaled-Distance models fit poorly. Model results were relatively unchanged with alternative prior distributions and likelihood functions (see Fig D in S1 Text). A qualitative comparison of the different models in the style of Fig 3B and 3C is shown in Fig C in S1 Text. The comparison reveals that the Heuristic model, Ideal-Confidence-Observer model, and the Unscaled-Distance model have distinct patterns of confidence choice-probabilities.

**Table 3:** Equations for the seven base Type 2 Confidence models. The models consider the decision evidence, Ev(), in favour of the perceptual choice, *r*, given the dot measurements, *X*, and Type 1 decision criterion, *k*_1_. The DFC Evidence-Strength models take a point estimate of the posterior’s mode ($\hat{\mu }$), with or without scaling by the posterior’s spread ($\hat{\sigma }$), and compare its unsigned distance from *k*_1_. Confidence forced-choice judgements involve comparing the relative confidence evidence for the intervals (*w*_1_ versus *w*_2_), with an influence of a confidence interval bias, *k*_2_.

Model	Confidence Evidence	Decision Rule	Confidence Noise	Free Parameters
Ideal Conf. Observer	*w* = Ev(*r*|*X*, *k*_1_)	*w*_2_ > *w*_1_	none	none
Basic Probability	*w* ∼ Beta(*νp*, *ν*(1 − *p*)); *p* = Ev(*r*|*X*, *k*_1_)	*w*_2_ + *k*_2_ > *w*_1_	0 < *ν* < ∞	*ν*, *k*_2_
Probability Difference	*w* = Ev(*r*_2_|*X*_2_, *k*_1_) − Ev(*r*_1_|*X*_1_, *k*_1_) + *ϵ*	*w* + *k*_2_ > 0	$\epsilon ∼N(0,\sigma_{\text{conf}}^{2})$	*σ*_conf_, *k*_2_
Log Probability Ratio	$w=|\text{log}\;(\frac{p}{1-p})+\epsilon |$ ; *p* = Ev(*r*|*X*, *k*_1_)	*w*_2_ + *k*_2_ > *w*_1_	$\epsilon ∼N(0,\sigma_{\text{conf}}^{2})$	*σ*_conf_, *k*_2_
Unscaled Distance	$w=|\hat{\mu }-k_{1}+\epsilon |$	*w*_2_ + *k*_2_ > *w*_1_	$\epsilon ∼N(0,\sigma_{\text{conf}}^{2})$	*σ*_conf_, *k*_2_
Scaled Distance	$w=|\frac{\hat{\mu }-k_{1}}{\hat{\sigma }}+\epsilon |$	*w*_2_ + *k*2 > *w*_1_	$\epsilon ∼N(0,\sigma_{\text{conf}}^{2})$	*σ*_conf_, *k*_2_
Heuristic	$w=\Delta |\mu_{c}-k_{1}|+\beta_{1}\Delta N-\beta_{2}\Delta \frac{1}{\sigma_{\text{emp}}}+\epsilon$	*w* + *k*_2_ > 0	$\epsilon ∼N(0,\sigma_{\text{conf}}^{2})$	*β*_1_, *β*_2_, *σ*_conf_, *k*_2_

### Examining the Heuristic model fits

We used the best-fitting parameters of the Heuristic model to investigate choice behaviour further. Fig 4C shows the estimated coefficients, which can be compared directly because the predictors were z-scored. Confidence was most strongly determined by the stimulus strength (i.e., dot-cloud position). Of the two sensory-uncertainty manipulations, the quantity of information (i.e., number of dots) was given more weight than the quality of the information (i.e., dot-cloud spread), as can be seen by the coefficients, *β*_*quantity*_ = 0.68 ± 0.10 versus *β*_*quality*_ = 0.38 ± 0.09 (*t*_15_ = 3.75, *p* < 0.01) respectively, which are also both significantly different from 0 (*t*_15_ = 6.61, *p* < 0.01 and *t*_15_ = 4.34, *p* < 0.01 respectively). These are contrasted per participant in Fig 4D. There appears to be clustering of coefficient values according to best-fitting model (examined in more detail in Fig E in S1 Text), with only Heuristic-best-fit participants giving more weight to dot quantity than stimulus strength. According to the best-fitting parameters for the Heuristic model across all participants, confidence noise was *σ*_*conf*_ = 1.20 ± 0.17 and there was a slight but not significant confidence interval bias to choose Interval 2 as more confident (*β*_*bias*_ = *k*_2_ = 0.13 ± 0.08; *t*_15_ = 1.63, *p* > 0.05; Fig 4C). In the confidence forced-choice paradigm, a confidence bias for one interval could indicate a memory effect (e.g., selecting Interval 2 more frequently because that decision is better remembered), so a non-significant confidence interval bias suggests our task was well-paced enough to avoid memory constraints in the confidence comparison. The reader can see the confidence interval bias of the other model fits in Table A in S1 Text.

We then examined if the Type 1 performance differed between the Heuristic best-fit participants and the others. The estimated sensory noise parameter *σ*_*dot*_ was higher in the Heuristic group versus the non-Heuristic group, 1.05 ± 0.04 versus 0.90 ± 0.06, but not significantly (*t*_14_ = 1.89, *p* > 0.05). For a model-independent way of comparing Type 2 performance between these two groups, we used the difference in accuracy between chosen and declined trials (Fig 3A). The Heuristic group had a significantly lower accuracy difference from the non-Heuristic group (7.94 ± 1.21% versus 10.70 ± 0.28%, *t*_14_ = −2.51, *p* < 0.05), reflecting lower metacognitive sensitivity.

### Model recovery analysis

A model recovery analysis further supported our model-comparison results. Data were simulated according to a particular model, with identical experiment structure and parameters consistent with our participants’ behaviour. Our simulated data were almost always best-fit by the model that generated them (Fig 4E). This indicates that the models are distinguishable from one another in model comparison. Thus, differences in the best-fitting model across participants likely reflects idiosyncrasies in the confidence computation, detectable due to the high number of trials per participant, rather than measurement noise one would expect in a low-powered design. The centred-prior variant of the Basic-Probability model had the lowest recovery success with 78.75% datasets recovered. The Heuristic model was the highest with a 100% recovery rate. On average the recovery rate was 91.72 ± 6.54% (mean ± SD).

A comparison of the relative AICc scores in Fig 4F shows that some model-simulation fit pairs are more similar in model-fit quality than others, even though the simulated model was almost always correctly recovered. First, flat- and centred-prior variants tended to have similar AICc scores. A similar pattern emerges for the Scaled-Distance and LPR models. Then there are the unidirectional similarities. Datasets generated by simulating the Ideal-Confidence-Observer model could often be well fit by the centred-prior variants of other models. This was to be expected if the model converges to the Ideal-Confidence-Observer model when confidence noise and interval bias approach 0. But, by penalising model complexity, the Ideal-Confidence-Observer model is often recovered. The other unidirectional similarity is between the Unscaled-Distance models and the Heuristic model. This is because the Heuristic model with no weight given to the quantity and quality predictors is the likelihood-variant Unscaled-Distance model. However, penalising the extra complexity of the Heuristic model helps to ensure the recovery of the Unscaled-Distance models.

### Confidence agreement

**Fig 5:**
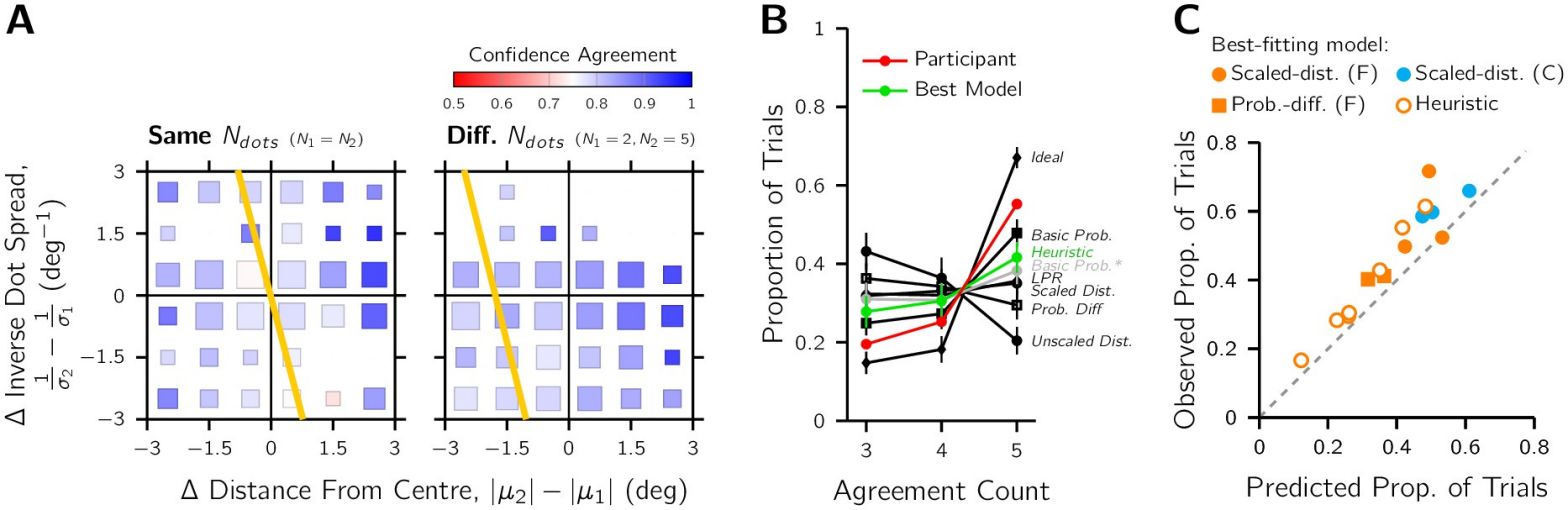
Confidence agreement results (*n* = 15). Confidence agreement was calculated per trial as the proportion of the most-selected confidence choice for the participants who did a 3-, 4-, or 5-pass version of the experiment (Fig 2B). A) Heatmaps of average confidence agreement according to the properties of the two stimuli displayed, pooled across observers. Gold: the indifference lines where each interval is equally likely to be selected as more confident according to the preliminary analyses (Fig 3B). B) Comparing the 5-pass confidence agreement of a representative example participant (red; #11) with the predicted confidence agreement of the models. Green: the best-fitting model for this observer (Heuristic model). Black: other models (flat- and centred-prior variants had similar confidence agreement counts so only the flat-prior variant is shown). Grey: the Basic-Probability model with additional late noise (1% SD). Model predictions calculated from 100 simulated datasets using the participant-specific best-fitting parameters. Error bars: ±2 SD. C) A comparison of the predicted and the observed proportion of trials for the highest agreement count. Each marker is an individual participant, where marker style indicates their best-fitting model (“F” refers to the flat-prior variant and “C” the centred-prior variant). The best-fitting model per observer was used for the confidence-agreement prediction. Dashed line of equality is also shown.

Confidence agreement was quantified by counting the number of the most consistent confidence response on a per-trial basis (e.g, 4 “Interval 1” responses out of 5 passes is 80% agreement). The pattern of confidence agreement averaged across observers shows that 1) observers had high overall levels of confidence agreement, and 2) comparisons close to the confidence-indifference line are less consistent than those far from this line (Fig 5A). We then investigated the predicted confidence agreement according to each of the twelve models, simulated using the best-fitting parameters on a per-participant basis (results of an example participant shown Fig 5B and all participants in Fig F in S1 Text). As expected, the Ideal-Confidence-Observer model always had the highest confidence agreement, because there is no confidence noise in this model. The Basic-Probability models then had the second and third highest levels of confidence agreement, followed by the remaining models, which tended to have more similar levels of confidence agreement. The higher confidence agreement of the Basic-Probability models, however, was not robust. As expected, many of the confidence comparisons had values close to 1 (Fig 1D), with the computer simulations detecting very small differences in probability (e.g., 0.985 versus 0.998). A human observer is unlikely capable of such comparisons, and so to get a more realistic prediction of confidence agreement for the Basic-Probability model, we also simulated a version of the model that included 1% SD late noise (grey lines in Fig 5B). The confidence agreement of this model was much closer to the other non-ideal models.

To investigate if the best fitting model captured the confidence agreement of observers, we compared the proportion of trials for the highest possible agreement count. Note that the minimum and maximum agreement depended on whether the participant did a 3-, 4-, or 5-pass version of the task. As the minimum number of passes was 3 (e.g., 2/3 and 3/3 agreement), selecting only a single count value to compare was the only appropriate choice for a statistical comparison. For 14 of the 15 observers, their confidence behaviour was in closer agreement across the repeats than predicted by their best-fitting model (see Fig 5C). A Wilcoxon signed-rank test confirmed that this difference in confidence agreement is significant (*n* = 15, *z* = 3.35, *p* < 0.01).

## Discussion

We fit confidence models of all three metric types to confidence forced-choice responses in a suprathreshold spatial-discrimination task. Each metric type was supported by at least one observer, with 44% observers best fit by the Heuristic model (Heuristic metric), 44% by a Scaled-Distance model (DFC Evidence-Strength metric with a SNR transformation), and 12% by the Probability-Difference model (Probability metric with late confidence noise). The Heuristic model was considered the winner overall at the group level. The modelling results suggest there is no universal computational strategy for taking sensory uncertainty into account when judging confidence. All four possibilities (i.e, a probability metric, SNR-scaling, use of a centred-prior, heuristic cue use; see Fig 1) were supported by at least one observer. Taken together, these results suggest that the computation of confidence is highly idiosyncratic in environments where decision uncertainty is influenced by multiple factors, further supporting the hypothesis of a highly individual nature to perceptual confidence [42–44], which may also depend on the task at hand [32, 35]. However, within the metric types, there was a preference for some computations over others, which has implications for our understanding of the computation of perceptual confidence more generally.

### Evidence-Strength metrics

The Unscaled-Distance model performed significantly worse than the Scaled-Distance model, which took into account sensory uncertainty by applying a SNR transformation to the point estimate used to compute the DFC. In other words, a single point estimate of the decision process was insufficient to capture confidence behaviour and a secondary point estimate of sensory uncertainty is used by the metacognitive system. This finding is particularly relevant for the Extended-SDT framework of perceptual confidence. It is often assumed that the sensory measurement is simply compared to static confidence criteria [6, 22], which is no issue due to many SDT tasks being of a fixed difficulty level (note that this is not true of tasks that staircase difficulty, e.g., [25, 45]). In mixed-difficulty designs, however, it has been proposed that confidence criteria are updated according to the level of sensory uncertainty [11, 27, 46]. The reason an observer would do this is to avoid a confidence paradox of more readily assigning high confidence to stimuli with large sensory noise that are more likely to have the measurement fall far from the perceptual decision criterion. Yet, human observers do not shift their criteria appropriately to avoid this paradox [27, 46]. However, without an incentive structure for confidence, confidence ratings are essentially meaningless and there is little motivating accurate shifts, which could explain these results. Though this is not true for the confidence forced-choice judgements as relative comparisons will always have a sensible interpretation even in the absence of an incentive structure.

From a modelling perspective, shifting the criteria to account for sensory uncertainty and the scaled DFC measure have the same effects on the confidence computation. Previous studies likely favoured the shifting-criterion description simply because criterion plasticity is commonly accepted [47], whereas in the current study there were no confidence criteria applied to the confidence decision variable because the two confidence decision variables are directly compared [41]. Our results suggest shifting of confidence criteria may not be necessary as observers can scale DFC measures, however the “stickiness” of criteria has been useful for explaining suboptimal behaviour in decision contexts requiring shifting criteria [27, 48, 49]. To explain suboptimal confidence judgements in this manner within the confidence forced-choice paradigm, one would need to apply noise to the interval bias term (i.e., *k*_2_). However, we interpret a changing propensity to report one interval over another as distinct from traditional criterion biases, which reflect under- and over-confidence. This highlights the need to better understand the metacognitive mechanism for accounting for uncertainty. If researchers wish to apply SNR scaling to an Extended SDT model, we propose using the log-likelihood-ratio representation (ln *β*), which rescales the decision axis by the amount of sensory noise for a given stimulus level [50], as placing perceptual and confidence criteria in this space removes the need to model multiple shifted criteria.

### Heuristic metrics

The Heuristic model in this task had to consider three stimulus-based cues when assigning confidence: the position of the dot cloud, the number of dots, and the dot spread. The distance of the centroid from the centre was the predictor given the most weight, followed by the number of dots, then dot spread. Heuristic observers tended to give more weight to the number of dots displayed than the other observers, with 5/7 observers giving almost equal or more weight to this predictor than the position of the dot cloud (i.e., stimulus strength). It is unclear why the number of dots was particularly salient to these observers in this task, given the amount of previous evidence suggesting that variability, here dot-cloud spread, is a strong heuristic cue [14, 33–35]. However, to our knowledge, no other perceptual confidence task has jointly manipulated both the quantity and quality of sensory information. This suggests that future work interested in investigating heuristic cues in confidence should consider targeting the quantity of information provided.

From a computational perspective, it may not always be possible to distinguish a Heuristic observer from an Evidence-Strength observer. Take, for example, a task that only manipulates stimulus strength. With the same noise sample, the estimate of stimulus-strength magnitude by the Heuristic observer is identical to the Evidence-Strength observer’s distance-from-criterion metric as long as the observer is unbiased. Thus, despite different underlying process models, these two observers are likely to display similar confidence behaviour. This could be seen in our model recovery analysis, where simulations of the Unscaled-Distance model (which only considered stimulus strength) could be somewhat well fit by the Heuristic model, though penalising model complexity almost always led to the correct model being recovered. As such, manipulations of sensory uncertainty help disambiguate these metric types, as will strong biases in the Type 1 criterion (e.g., by manipulating stimulus priors or rewards, see [21]). However, varying sensory uncertainty is only helpful if the Evidence-Strength model is constrained appropriately, either by principally relating the relevant stimulus factors to the likelihood uncertainty as we did here, or enforcing the corresponding relationship to the d’ of different difficulty levels for Extended-SDT. Otherwise, if likelihood uncertainty or d’ values are fit independently for each unique difficulty level, the Evidence-Strength and Heuristic models may appear to fit more similarly.

### Probability metrics

In addition to the Probability-Difference model, which best-fit two observers, the Probability metric category contained the Ideal Confidence Observer, the Basic-Probability model, and the LPR model. The centred-prior variants of these models conform to the definition of “Bayesian Confidence” according to the Bayesian Confidence Hypothesis [10–12], and were not a best-fitting model of any participant. It is unclear if the flat-prior variants of these models also conform to the definition of Bayesian Confidence, which is unspecific about whether a true stimulus prior is required. Overall, our results do not support the Bayesian Confidence Hypothesis, in agreement with some previous studies [11, 28, 39] but not others [12, 32]. Despite this finding, the results of the current study should not be interpreted as against Bayesian computations in the brain more generally. The averaging of the perceived dot locations that is the basis of all models we tested is optimal Bayesian cue combination [51]. Additionally, the computation of the posterior mode in the centred-prior variants of the Unscaled- and Scaled-Distance models must also involve a Bayesian computation. This is ambiguous in the case of the flat-prior variants of Unscaled- and Scaled-Distance models, because these models are identical to a model that uses the point estimate from the un-normalised likelihood function.

Another consideration in the computation of confidence is the resolution of probability judgements for confidence evaluations. This can be best seen in the contrast of the Basic-Probability model and the LPR model. If the distribution means in both intervals are far from the centre, the raw probability values will be similarly close to 1 (Fig 1D), but if a LPR transformation is applied, small differences in the relative positions of the distribution means can translate into large differences in confidence (Fig 1E). Effectively, the LPR model has a very high resolution for extreme probability comparisons. Fortunately, this can be assessed in the confidence forced-choice method despite potential distortions in perceived probability [52, 53] because any distortion would apply to both Interval 1 and 2 and thus not change the relative judgement in confidence, as long as the distortion function is monotonic. In contrast, traditional methods of measuring confidence are likely to suffer from probability distortions in probability judgements or ceiling effects of ratings at high levels of confidence [20]. Our results are not conclusive on this point. While the Probability-Difference model was third best in terms of the number of observers best fit, the LPR model was overall a superior model at the group level and in the model recovery analysis showed some fit similarity to the second best-fitting Scaled-Distance model (Fig 4F). Reviewing previous studies, a high resolution for extreme probabilities does not match with findings of compression of continuous confidence probabilities to a few discrete levels for perceptual confidence [39] or knowledge of motor uncertainty distributions (i.e., motor confidence, [54]). Further work is needed to understand the representation of confidence for extreme probabilities. It is possible that our Probability-metric models were overly simplistic, and models with intermediate resolution, such as by allowing the confidence noise to vary with signal strength or including probability distortions in the mapping function [55], would improve the fit. However, the increased flexibility of such models is also likely to pose a challenge in distinguishing between candidate models.

### Use of a centred prior

We were unable to reach a strong conclusion about the use of centred priors in the computation of confidence. Only three observers were best-fit by a centred-prior variant model (Fig 4B), but this increased to six if the stimulus prior assumed perfect knowledge of the three levels of dot spread tested (see Fig D in S1 Text). It is well known that observers do not always adapt perfectly to the experimental environment, causing them to use incorrect priors or priors matching environmental statistics [56, 57], and this may have occurred in the current study.

### Heterogeneity in the confidence computation

We found a variety of computational strategies in our sample population. The model-recovery results support the conclusion of model heterogeneity [58, 59] against other possible interpretations, such as high measurement noise. Our results are consistent with the previously reported heterogeneity in confidence modelling, within and across studies [11, 12, 32, 35, 39]. We consider the possibility that task demands influence the adopted strategy generally and in the present study.

First there is the use of mostly suprathreshold Type 1 decisions paired with the confidence forced-choice reporting method. The Heuristic and Evidence-Strength metrics are both good strategies for avoiding confidence indifference in the comparison of two certain choices. This is because often one dot cloud will be further from the centre, have more dots, or a smaller spread. In contrast, comparing near-ceiling probabilities of being correct is more likely to lead to indecision or indifference, which could make the participant feel like they are not doing well in the task. Thus, our task may have influenced the adoption of a specific strategy. However, it is important to consider that suprathreshold decisions and confidence forced-choice are not rare but a common part of real-world decision-making and should be considered for a complete description of confidence behaviour. For example, when one is in the supermarket attempting to select the freshest salad mix of two already decently fresh options, the Heuristic approach of selecting the bag without spinach because it’s known to expire quickly will lead to a satisfactory result fast. Broadly, it would be advantageous to be able to switch from more optimal computations to something that can provide a quicker and less complex answer if the situation called for it [60]. The second concern regards the complexity of the confidence report method itself. A misconception about the confidence forced-choice method is that it is more difficult for the decision-maker than single-trial methods for reporting confidence (e.g., ratings on discrete or continuous scales). Participants in a confidence forced-choice paradigm do have to hold in memory the confidence of the previous choice for the comparison. However, it is unclear if this is more taxing for the participant than keeping in memory multiple confidence criteria [6, 27] or a confidence-response mapping function [35, 61]. Thus, further research would be required to claim method-complexity as a reason for adopting strategies that make use of Heuristic or Evidence-Strength metrics.

If multiple confidence strategies are possible, across or within observers, what then should be the goal of the computational analysis? One important result would be to scope out the general limitations of the metacognitive system. For example, does the metacognitive system have access to the full posterior distribution? Our results suggest this may be possible (i.e., the Probability-Difference observers), but the use of mean and uncertainty point estimates were more common (i.e., the Scaled-Distance observers; also reported by [11]). What is clear is that answering this question involves testing large sets of distinguishable models in a range of decision contexts. Such testing could also be used to construct a systematic description of contextual effects on confidence strategy (e.g., choice difficulty, mixed-uncertainty environments, reward structure, attentional resources, etc.). Combining a multi-model approach with neural measures could also be fruitful for understanding the neural activity associated with the different confidence strategies [57]. For example, a classifier that could independently partition observers according to similar patterns of neural activity could then be compared to the grouping of observers by model best-fit, which could serve as a test of the strategy-heterogeneity hypothesis. Similarly, if activity in one particular area of the brain is correlated with a particular model parameter, we would expect the observers better-fit by another model to show a weaker relationship between that parameter and neural activation. Importantly, the multi-strategy framing of confidence could help researchers avoid unproductively attempting to reach a consensus as to the single model of confidence, if indeed humans employ more than one strategy.

### Confidence agreement

The confidence agreement analysis showed that the best-fitting model per observer almost always underestimated the degree of confidence agreement in the N-pass designs. From a modelling perspective, there are three factors that could have limited confidence agreement in the present experiment: 1) Type 1 sensory noise, 2) Type 2 confidence noise, and 3) the resolution of the confidence decision variable. The effect of sensory noise can be observed in the predicted confidence agreement of the Ideal Confidence Observer (Fig 5B), as this was the only factor relevant in the simulation of this model. The remaining models have lower predicted confidence agreement, which is due to the influence of the confidence noise. The Basic-Probability model had higher confidence agreement than the others, likely due to the model using a different noise distribution (Table 3). Examining the model simulations in more detail, we found that many of the confidence comparisons for the Basic-Probability model were between two extremely high probabilities and that the model is not robust to even small amounts of late decision noise. It is unlikely that a human observer has such a high resolution for the confidence decision variable, which is why resolution may be a third factor, effectively discretising confidence into various levels [39]. However, lowering the confidence resolution will decrease confidence agreement, so this alone cannot explain why the confidence models with non-discretised confidence variables under-predicted confidence agreement. It is also not due to an interval response bias, as this was included in the model simulations.

What the Basic-Probability model does illustrate is that the choice of the confidence noise model affects confidence agreement. This suggests that future studies of confidence agreement could investigate different confidence-noise distributions [61, 62], assumptions about noise being independent between the two intervals, partially or fully parallel confidence decision processes [7], and serial-dependence effects on confidence [63]. In general, the N-pass technique offers an interesting additional benchmark for assessing confidence models, just as it has proved useful in better understanding the computations of perceptual decision-making [64–66]. It is important to keep in mind that the inputs to the confidence computation will never be identical due to sensory noise, so not all analyses developed for perceptual decision-making will be applicable to their confidence counterpart. Overall, confidence agreement appears to be a promising evaluation technique, with some researchers already leveraging the power of multiple presentations mostly in the form of trial “replays” to understand confidence [31, 67], so we expect that the use of multiple passes in confidence experiments will only increase.

### Strengths and limitations

A key strength of this study was the variety of metric types tested. We succeeded in fitting models of the Probability, Evidence-Strength, and Heuristic types while maintaining reasonable model identifiability (see the model recovery analysis in Fig 4E). In part, this was due to our novel approach of pairing the confidence forced-choice technique [26, 41] with an easy perceptual task of sufficient complexity. Specifically, the models are more divergent for easy trials and the confidence forced-choice method allowed us to probe the confidence decision variable in this range.

A downside of the confidence forced-choice technique is that there is only one confidence report for every two perceptual decisions, doubling the number of perceptual trials needed per participant. In our study, participants completed five hours of testing each on trials with very brief stimulus presentations. Using stimuli that require long presentation times (e.g., random-dot motion) would have a serious impact on the data collection rate and introduce concerns over memory of the decision in the first interval. A consequence of using very brief stimulus presentations is that these stimuli are arguably less suitable for studying the accumulation of evidence. Typically, researchers who investigate the temporal dynamics of decision-making with accumulation-to-bound models use long presentation times or let the observer decide when to terminate viewing the stimulus [5, 30, 31]. For these reasons, we did not investigate accumulation-to-bound models in the present study, despite their better-established link to the neural basis of decision-making [29]. A consequence of this choice is that we neglected to measure the decision reaction times in our study, which in hindsight could have been a relevant cue in the Heuristic model. Future work could contrast accumulator metrics versus Heuristics and DFC-Evidence-Strength metrics. We highlight that very recent research on this topic [62] indicates that the two-stage accumulator model [8] does not out-compete the DFC Evidence-Strength model [61].

The unintentional repetition of sessions resulting in an N-pass design to the experiment had both strengths and limitations. Obvious concerns are a reduction in the number of unique stimuli and stimulus pairings in the task, which affects the quality of the dataset for model fitting. There is also the possibility of the observer recognising the repetition and repeating responses, though none reported noticing the N-pass nature of the task. The clear advantage was the ability to conduct an exploratory analysis of confidence agreement, which revealed an explanatory deficit in the best-fitting models. We recommend researchers in confidence agreement design the N-pass carefully. For example, one can avoid order effects by shuffling the order of repeated pairs [66]. However, with the repeated-sessions technique accidentally employed here, the precise sequence of stimulation was identical, as was the position of the pair within the session. This could keep constant stimulus history effects [68, 69], response history effects to some extent in the case of a suprathreshold task, and fatigue or motivation changes during the session. However, the researcher would run the risk of the observer noticing the repetition, leading to response memorisation or surprise effects, if the number of trials was low. Regarding motivation or surprise effects, these are less of a concern for the confidence forced-choice method with exactly repeated sets as these effects should apply to both Interval 1 and 2 almost equally. Another consideration is the difficulty of the task. Difficult trials are more likely to lead to Type 1 judgements that differ between passes, whereas in easier designs, such as in the present study, the majority of Type 1 judgements are the same. Finally, we see no reason why confidence-agreement experiments cannot be used with other types of confidence reports besides confidence forced-choice. As such, including at least a 2-pass design into any confidence experiment should be feasible.

## Conclusion

By using the confidence forced-choice method, we have shown that observers are not indifferent for easy perceptual choices. Almost half of the observers took sensory uncertainty into account by computing the signal-to-noise ratio (SNR) in the Type 1 decision process, while a similar number used stimulus-based heuristic cues to compute confidence. Overall, this suggests that observers use the Distance-From-Criterion (DFC) Evidence-Strength and Heuristic metrics over Probability metrics, the most notable of which follow the Bayesian Confidence Hypothesis. Furthermore, while heuristic cue use is likely to vary according to the specifics of the individual experiment, the Scaled-Distance model is applicable in any mixed-difficulty design and should be more widely considered. Our results suggest that relying on the simple unscaled metric typically used in extended Signal Detection Theory (SDT) could lead to worse model fits or an unnecessary proliferation of confidence criteria to account for this transformation. An accidental repetition of the presented stimuli also allowed us to capture the confidence agreement of observers; a novel measure of model fit. This analysis revealed that observers are much more consistent than would be predicted by any of the models except the Ideal Confidence Observer. We propose confidence agreement as a readily accessible model-validation benchmark for future efforts in modelling perceptual confidence.

## Materials and methods

### Ethics statement

This study was approved by the New York University Committee on Activities Involving Human Subjects (IRB-FY2016–595). All participants received details of the experimental procedures and provided written consent prior to participation.

### Participants

Sixteen participants (21–43 years old, ten female) with normal or corrected-to-normal vision took part in the study. All participants but two were naive to the design of the experiment.

### Apparatus

Stimuli were displayed on a Sony G400 CRT monitor (36 x 27 cm, 1024 x 768 pixel, 85 Hz). Participants sat 55 cm from the monitor with their head stabilised by a chin rest. All responses were entered on a standard computer keyboard. The experiment was conducted using custom-written code in MATLAB version R2014a (The MathWorks, Natick, MA), using Psychtoolbox version 3.0.11 [70–72].

### Task

In this task, participants judged if the mean of an invisible dot-generating distribution, a 2D circular symmetric Gaussian, was left or right of centre (i.e., a *Type 1*, perceptual judgement). The distribution had seven possible spatial offsets of the mean (-4, -2, -1, 0, 1, 2, and 4 deg) and three possible standard deviations (1.5, 2, or 2.5 deg). The distribution mean did not deviate vertically from the half-height of the screen and the screen centre was indicated by a fixation cross prior to stimulus presentation. Participants were presented with either two or five independently sampled dots (Gaussian-blobs with 0.1 deg SD). The white dots were simultaneously presented on a mid-grey background for 23.5 ms. The number of dots (i.e., *quantity* manipulation) and the spread of the generating distribution (i.e., *quality* manipulation), produced six levels of sensory uncertainty. Fewer dots or larger spread made the distribution mean more difficult to localise. After every two stimulus presentations (denoted Interval 1 and Interval 2), the participant reported if they had greater confidence that their first decision or second decision was correct (i.e., a *Type 2*, metacognitive judgement). This confidence forced-choice technique avoids over- or under-confidence biases by having the observer report relative confidence [26, 41]. An example trial pair is shown in Fig 2A. The stimulus location, number of dots, and distribution spread were randomised at the level of individual trials in an interleaved design, with trial pairings left to chance. Consequently, confidence comparisons could be between any combination of stimulus strength and sensory uncertainty (42 × 42 possibilities). In each session, there were 20 presentations per unique combination of stimulus strength and sensory uncertainty. New dots were sampled for each repeat. Participants completed 5 one-hour sessions of 840 perceptual judgements and 420 confidence forced-choice judgements, resulting in a total of 4200 and 2100 judgements, respectively, per participant. During the experiment, no feedback was provided on the correctness of the perceptual decisions. Data from this experiment are available at https://osf.io/k2nhq/.

### Stimulus set repetition

Due to a coding oversight, the experiment was conducted with the same random seed for every session. This, coupled with the practice of switching off the testing computer between sessions, led to many of the sessions displaying the exact same sequence of stimuli (Fig 2B). For example, eight participants saw the same identical sequence of dot clouds for all five sessions; three saw this sequence for four out of five sessions; and only one participant was given a unique stimulus set for each session. In the most frequent stimulus set, of the possible pairings of sensory uncertainty for the confidence comparison, the worst sampled combination had seven unique pairs of stimuli. If the interval order is ignored, this number increases to nine unique pairs of stimuli for two same-uncertainty pairings. In the experiment debriefing, no participant reported noticing the repetition of stimulus sets. While this random seed setting limited the richness of the collected dataset, we saw this as an opportunity to investigate the agreement of confidence reports for exactly identical dot-cloud stimuli and thus identical forced-choice comparisons. In our modelling, we were able to leverage these measurements of confidence agreement to perform predictive checks of the model fits.

## Models

### General modelling framework

**Fig 6:**
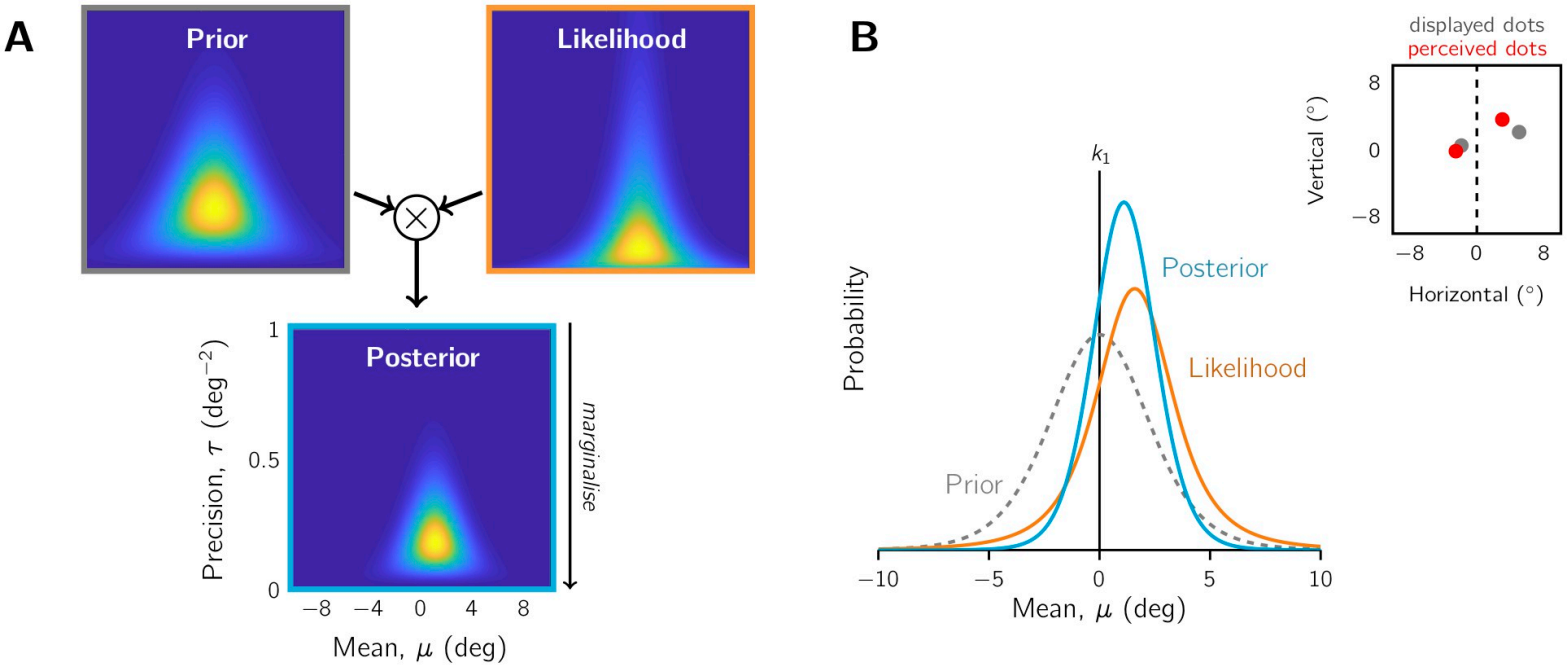
Elements of the decision models. Depicted is the estimated joint probability of the dot-cloud generating distribution mean and precision for an example stimulus presentation of 2 dots. A) Centred Normal-gamma prior distribution (based on the stimulus statistics of the experiment), likelihood function based on the noisy dot observations, and the resulting normal-gamma posterior distribution. All three distributions have the same axes as the ones shown in the posterior panel. B) The marginal prior (grey), likelihood (orange), and posterior (blue) for estimating the generating distribution mean. An unbiased Type 1 decision criterion, *k*_1_, is also depicted. Inset: the displayed and perceived locations of the two sampled dots, with the dashed line indicating the screen midline.

We modelled our participants as Bayesian observers making a joint inference about the mean and precision of the dot generating distribution from a noisy observation of the dots presented onscreen. Fig 6A shows the prior, likelihood, and posterior components for an example trial. It was important to include the inference about the precision of the generating distribution because uncertainty in the estimated mean depends on the precision (note that precision is the inverse variance, 1/*σ*^2^). This can be seen in the triangular shape of the prior, likelihood, and posterior. If the distribution’s spread is small, precision is large, and the uncertainty in the distribution’s mean is low. To make a spatial judgement, the observer marginalises over all possible precision values to get the probabilistic representation shown in Fig 6B. From this they decide whether the evidence favours a distribution mean to the left or the right, and subsequently their confidence. The advantage of using the same general framework for models of all three metric types (Probability, Evidence-Strength, and Heuristic) was that conclusions drawn from the model fits were more likely to reflect the confidence computation than “nuisance” differences between different modelling frameworks.

### Decision context

There are two categories of stimuli in the perceptual task: left (*C* = *L*) and right (*C* = *R*). For each interval, the observer reports their belief about stimulus category (i.e., $\hat{C}=L$ or $\hat{C}=R$), as their Type 1 response based on their noisy sensory measurements of the sampled dots as well as any prior beliefs about the underlying generative sensory process. For the Type 2 confidence judgement, the observer compares the probability of their Type 1 perceptual decisions being correct in Interval 1 and 2: $p({\hat{C}}_{1}=C_{1})$ versus $p({\hat{C}}_{2}=C_{2})$.

In a single interval, a dot sampling distribution, defined by its horizontal mean (*μ*_*cloud*_) and spread (*σ*_*cloud*_), is drawn from one of the respective categories with equal probability: *P*(*C*_*L*_) = *P*(*C*_*R*_) = 0.5. The exception being the case where *μ* = 0, which favours neither category and we treat as coming from either category randomly. From the sampling distribution, *N* dots are independently drawn, represented by the vector of horizontal dot locations, ***D*** = *d*_1_, *d*_2_, …, *d*_*N*_. During the measurement process, additive sensory noise is applied per dot, $\epsilon ∼N(0,\sigma_{dot}^{2})$, represented by the vector of noisy horizontal dot-location measurements, ***X*** = *x*_1_, *x*_2_, …, *x*_*N*_. We assume the observer knows *N* (either 2 or 5) but not *σ*_*cloud*_ of the sampling distribution on any given trial. Thus, the observer’s Type 1 task is to infer the category of stimulus with uncertain sampling distribution mean and spread based on the observed dot samples.

### The likelihood function

The observer must consider two sources of uncertainty when choosing a likelihood function, the noisy draws of *N* dots from the sampling distribution, affected by $\sigma_{cloud}^{2}$, and the internal noise applied to each dot, $\sigma_{dot}^{2}$. These two noise sources are additive, resulting in the combined precision per dot of

$$\begin{array}{c} \tau_{comb}=\frac{1}{\sigma_{cloud}^{2}+\sigma_{dot}^{2}}=\frac{1}{\tau_{cloud}^{-1}+\tau_{dot}^{-1}}. \end{array}$$ (1)

Note that we have parameterised the variances in terms of precision: $\tau_{cloud}=\sigma_{cloud}^{-2}$ and $\tau_{dot}=\sigma_{dot}^{-2}$. The likelihood function for the observed dot cloud is

$$\begin{array}{cc} p(X|\mu_{cloud},\tau_{cloud},\tau_{dot}) & =\prod_{i=1}^{N}p\left(x_{i}|\mu_{cloud},\tau_{comb}\right)\\ & \propto \tau_{comb}^{\frac{N}{2}}\;\text{exp}\;(\frac{-\tau_{comb}}{2}\sum_{i=1}^{N}{\left(x_{i}-\mu_{cloud}\right)}^{2}). \end{array}$$ (2)

This likelihood function is centred on the dot-cloud centroid, the average of all the dot locations, because each dot is considered to be an equally reliable cue to the location of the generating distribution. An example likelihood function is shown in Fig 6A.

### The prior distribution

In the centred-prior variants, we assumed the observer correctly inferred the joint distribution of the sampling distribution mean and precision through experience with the task. Even though the mean and precision were discretised in our experiment (7 and 3 possible values, respectively), we assumed that the observer could not have such a detailed representation and thus we consider that the prior is a continuous distribution. The conjugate prior for our likelihood function (i.e., inferring a normal distribution with unknown mean and precision) is a normal-Gamma distribution [73, 74]:

$$\begin{array}{cc} p(\mu_{cloud},\tau_{cloud}) & =NG(\mu_{cloud},\tau_{cloud}|\mu_{0},\kappa_{0},\alpha_{0},\beta_{0})\\ & =N\left(\mu_{cloud}|\mu_{0},{\left(\kappa_{0}\tau_{cloud}\right)}^{-1}\right)\;Ga\left(\tau_{cloud}|\alpha_{0},\beta_{0}\right). \end{array}$$ (3)

To find the parameters of the normal-Gamma prior, we calculated the values of *μ*_0_, *κ*_0_, *α*_0_, and *β*_0_ such that the marginal means and variances matched the true stimulus statistics from the experiment (derivation in S1 Text). This prior distribution, shown in Fig 6A, has *μ*_0_ = 0, *κ*_0_ = 0.68, *α*_0_ = 3.84, and *β*_0_ = 13.48. Note that *κ*_0_ can be interpreted as a number of pseudo-observations, *α*_0_ as related to degrees of freedom, and *β*_0_ as related to the prior belief about pooled variance.

For the flat-prior variants, we applied a uniform distribution over all possible mean and spread values. The posterior distribution is thus equivalent to the normalised likelihood function. For the posterior distribution we detail next, we are referring to the centred-prior variant models.

### The posterior distribution

Using Bayes’ theorem, we can write the expression for the posterior that combines the prior beliefs about the joint probability of the distribution’s mean and precision with the observed dot evidence. Because we have used the conjugate prior, the posterior is also a normal-Gamma distribution (derivation provided in S1 Text):

$$\begin{array}{cc} p(\mu_{cloud},\tau_{cloud}|X,\tau_{dot}) & \propto p\left(X|\mu_{cloud},\tau_{cloud},\tau_{dot}\right)p\left(\mu_{cloud},\tau_{cloud}\right)\\ & =NG(\mu_{cloud},\tau_{cloud}|\mu_{p},\kappa_{p},\alpha_{p},\beta_{p},\tau_{dot}), \end{array}$$ (4)

with the following posterior parameters:

$$\begin{array}{c} \mu_{p}=\frac{\kappa_{0}\tau_{cloud}\mu_{0}+N\tau_{comb}\bar{x}}{\kappa_{0}\tau_{cloud}+N\tau_{comb}}, \end{array}$$ (5)

$$\begin{array}{c} \kappa_{p}=\frac{\kappa_{0}\tau_{cloud}+N\tau_{comb}}{\tau_{cloud}}, \end{array}$$ (6)

$$\begin{array}{c} \alpha_{p}=\alpha_{0}+\frac{N}{2}, \end{array}$$ (7)

and

$$\begin{array}{c} \beta_{p}=\beta_{0}+\frac{N}{2\tau_{cloud}}(\frac{\kappa_{0}\tau_{cloud}\tau_{comb}{\left(\bar{x}-\mu_{0}\right)}^{2}}{\kappa_{0}\tau_{cloud}+N\tau_{comb}}+\tau_{comb}s^{2}+\text{log}\;\left(\tau_{dot}^{-1}\tau_{cloud}+1\right)). \end{array}$$ (8)

Where $\bar{x}$ is the sample mean of the observed dots,

$$\begin{array}{c} \bar{x}=\frac{1}{N}\sum_{i=1}^{N}x_{i}, \end{array}$$ (9)

and *s*^2^ is the observed sample variance in maximum-likelihood terms,

$$\begin{array}{c} s^{2}=\frac{1}{N}\sum_{i=1}^{N}{\left(x_{i}-\bar{x}\right)}^{2}. \end{array}$$ (10)

An example posterior distribution is shown in Fig 6C.

### Taxonomy of confidence models

The seven base confidence models we considered differ in the level of access to the posterior distribution. They could access either 1) the full distribution, 2) only the mode, or 3) both mode and spread. Models that made use of the full distribution used Probability-metrics for computing confidence. Confidence computations that used the mode, with or without posterior spread, were DFC Evidence-Strength metrics. The Heuristic model combined separate estimates of stimulus strength and sensory uncertainty factors (i.e., number and spread of dots) in an idiosyncratic weighted sum. Model summaries are provided in Table 2 and model equations in Table 3.

We also considered two model variants when relevant, resulting in a total of twelve unique models. For the *centred-prior* variant, the observer uses the informative prior that matched the stimulus statistics (depicted in Fig 6). For the *flat-prior* variant, the observer uses a non-informative flat prior as if they had used the normalised likelihood function to make their perceptual and confidence decisions.

In regards to confidence interval bias and noise, as per its definition, the Ideal-Confidence-Observer model did not have either of these elements [41], but otherwise all confidence models had both. Confidence interval bias in the confidence forced-choice method is a preference for reporting Interval 2, for example and should not be confused with an over- or under-confidence bias [26, 41]. Confidence noise was dependent on the individual confidence model. Almost all models had additive Gaussian noise. However, for models that used raw probability values (i.e., the Basic-Probability models), we used Beta-distributed variability to keep the confidence decision variable between 0 and 1.

### Ideal-Confidence-Observer model

The goal of the observer was to infer if the mean of the dot sampling distribution was left or right of centre. The ideal confidence observer would take into account the unknown precision of the sampling distribution by marginalising over all possible precision values

$$\begin{array}{c} p\left(\mu_{cloud}|X\right)=\int p\left(\mu_{cloud},\tau_{cloud}|X,\tau_{dot}\right)d\tau_{cloud}, \end{array}$$ (11)

which results in the marginal posterior distribution shown in Fig 6B. The evidence for each category, given the observed measurements and centred stimulus prior, can be computed by

$$\begin{array}{c} p\left(C=R|X,k_{1}\right)=p\left(\mu_{cloud}>k_{1}|X,k_{1}\right)=\int_{k_{1}}^{\infty }p\left(\mu_{cloud}|X\right)d\mu_{cloud}, \end{array}$$ (12)

where *k*_1_ is the Type 1 discrimination boundary, and

$$\begin{array}{c} p\left(C=L|X,k_{1}\right)=1-p\left(C=R|X,k_{1}\right). \end{array}$$ (13)

For the Type 1 judgement, the inferred category of the stimulus, $\hat{C}$, is then determined by the relative probabilities of each category, with the observer selecting the most likely category. They select “right” if *p*(*C* = *R*|***X***, *k*_1_) > *p*(*C* = *L*|***X***, *k*_1_), and “left” otherwise. The observer reports the more likely category with a keypress ($r=\hat{C}$), unless a lapse occurs, where the observer selects the unintended category, with the lapse rate of λ. As such,

$$\begin{array}{c} p\left(\text{choose}\;\text{Right}\right)=\lambda +\left(1-2\lambda \right)p(p\left(C=R|X,k_{1}\right)>p\left(C=L|X,k_{1}\right)). \end{array}$$ (14)

For the Type 2 confidence judgement, the Ideal Confidence Observer considers the relative strength of the evidence in two consecutive Type 1 judgements, selecting the interval with the response that is more likely to be correct as having higher confidence, where Interval 1 evidence, *w*_1_, is

$$\begin{array}{c} w_{1}=\text{Ev}\left(r_{1}|X_{1},k_{1}\right)=\text{max}\;[p\left(C_{1}=R|X_{1},k_{1}\right),p\left(C_{1}=L|X_{1},k_{1}\right)] \end{array}$$ (15)

and Interval 2 evidence, *w*_2_ is

$$\begin{array}{c} w_{2}=\text{Ev}\left(r_{2}|X_{2},k_{1}\right)=\text{max}\;[p\left(C_{2}=R|X_{2},k_{1}\right),p\left(C_{2}=L|X_{2},k_{1}\right)]. \end{array}$$ (16)

The observer reports Interval 1 for the confidence judgement if *w*_1_ > *w*_2_ and Interval 2 if *w*_2_ > *w*_1_. When a lapse in the Type 1 report occurs, the observer selects the unintended response. We assume the participant is aware of the lapse and reflects this in their confidence report. Mathematically, the max operation in the above equations is replaced by a min operation for instances of lapses. The confidence evidence computation and decision rule are shown in Table 3, along with those for all Type 2 confidence models we tested. As this is the Ideal-Confidence-Observer model, the observer does not incur any additional metacognitive noise in their computation of confidence, but they are still subject to Type 1 decision bias through parameter *k*_1_ [41]. Following the decision rule of the ideal confidence observer,

$$\begin{array}{c} p\left(\text{choose}\;I_{2}\right)=\lambda +\left(1-2\lambda \right)p\left(w_{2}>w_{1}\right). \end{array}$$ (17)

We assumed the Type 2 lapse rate was identical to the Type 1 lapse rate, λ, and fixed it in the Type 2 model fits according to the best-fitting value from the Type 1 model fits. For both the Type 1 and 2 judgements, the choice probabilities were estimated by simulation. The Ideal-Confidence-Observer model had no free parameters after fixing the Type-1 parameters.

### Probability-metric models

The remaining six Probability-metric models follow a similar logic to the Ideal Confidence Observer at the Type 1 and 2 levels, but included various forms of metacognitive noise (Table 3). We also considered flat- and centred-prior variants. The posterior distribution in Eq 11 differs between the two variants, but otherwise the model computations are unchanged. In all of the Probability-metric models, we included a confidence interval bias term for interval preferences, which was implemented in the decision rule as −∞ < *k*_2_ < ∞. Thus, the Probability-metric models had two free parameters: *ν* or *σ*_*conf*_ (i..e., confidence noise), and *k*_2_.

#### Basic-Probability models

The observer directly compares the evidence in favour of their choice, but these raw probability values have been corrupted by noise. As probabilities are constrained to the interval [0, 1], we implemented a beta-noise model, with *ν* as a concentration parameter (larger values: less confidence noise). Effectively, *ν* is a number of “internal” observations, of which a certain fraction are consistent with the chosen category and the rest with the unchosen category, with proportions in line with the observer’s beliefs about being correct. Consequently, as *ν* → ∞, *w* becomes a Delta distribution at Ev(*r*|***X***, *k*_1_).

#### Probability-Difference models

The decision evidence is first compared for the intervals and then late additive Gaussian noise is applied to their difference. The smaller this confidence noise, *σ*_*conf*_, the closer the observer is to the ideal confidence observer.

#### Log-Probability-Ratio (LPR) models

The decision evidence of each interval is transformed onto a continuous scale of log probability and then Gaussian noise is applied before the intervals are compared.

### DFC Evidence-Strength metric models

The Distance-From-Criterion (DFC) Evidence-Strength models rely on point estimates from the decision process. In both the flat- and centred-prior variants, the observer computes the mode of the posterior, $\hat{\mu }$. Using this point estimate in the Type 1 decision will lead to identical Type 1 choice behaviour as in Eq 14, for the same prior variant. This is because a posterior mode right of the Type 1 criterion will always corresponds to *p*(*C* = *R*|***X***, *k*_1_) > *p*(*C* = *L*|***X***, *k*_1_) for the normal-gamma posterior distribution in Eq 4, and vice versa for a posterior mode to the left. However, the DFC Evidence-Strength models make different predictions for confidence. Similar to the Probability-metric models, we included a confidence interval bias, −∞ < *k*_2_ < ∞. Thus, the DFC Evidence-Strength models also had two free parameters: *σ*_*conf*_ and *k*_2_.

#### Unscaled-Distance model

For confidence evidence, this model considers the distance of $\hat{\mu }$ from the Type 1 discrimination boundary, *k*_1_, with added Gaussian noise, $N(0,\sigma_{conf}^{2})$, per interval. The further $\hat{\mu }$ is from the criterion, the stronger the evidence for the spatial discrimination judgement.

#### Scaled-Distance models

The observer also considers the decision uncertainty by assessing the spread of the marginal posterior distribution in the form of a second point estimate, $\hat{\sigma }$. The distance of the mode from *k*_1_ is then computed units of standard deviation (i.e., signal-to-noise ratio; see S1 Text for more details).

### Heuristic model

The *Heuristic* model uses an estimate of stimulus strength and estimates of each separate factor affecting sensory uncertainty (i.e, the number of dots and dot spread) as inputs to the confidence computation, without any constraint on the relative weighting of these inputs on confidence. In effect, this model is the best-fitting model from the preliminary logistic regression analysis (see Fig B in S1 Text), but fit in accordance with the discrimination behaviour (Table 3). This involved combining the noisy observations of the sampled dots to compute the position predictor (i.e., the centroid DFC |*μ*_*c*_ − *k*_1_|), the dot spread for the quality predictor (i.e., the empirical spread, *σ*_emp_), and a count of the number of dots for the quantity predictor (*N*), as well as any confidence interval bias (*k*_2_). To avoid equivalent best-fitting regressors, the position coefficient that was expected to be dominant was fixed at 1 in the model and the other two coefficients, *β*_1_ (for the difference in the number of dots across intervals) and *β*_2_ (for the difference in inverse dot spread), were free to vary. Confidence noise, *σ*_*conf*_, was also free to vary. In total, the Heuristic model had 4 free parameters: *β*_1_, *β*_2_, *σ*_*conf*_, and *k*_2_.

### Model fitting, comparison, and validation

Models were fit in a two-step procedure using custom-written Matlab code (available at https://osf.io/k2nhq/). Type 1 parameters were fit first (*σ*_*dot*_, *k*_1_, and λ) using the discrimination responses. Then the Type 2 parameters (*ν* or *σ*_*conf*_, *k*_2_, and possibly the two *β* values) were estimated using the confidence forced-choice responses. Type 1 parameters were kept fixed at their best-fitting values for the Type 2 fits. Type 1 models were fit using a brute-force grid method, with response probabilities estimated by simulation of the observer. For the Type 2 model fits, only simulated sensory measurements consistent with the observer’s Type 1 responses were used for fitting to ensure the calculated response probabilities were conditional on the discrimination choice. We used Bayesian Adaptive Direct Search (BADS) with the BADS toolbox [75], for the Type 2 model fits. Models were then compared in terms of their corrected Akaike information criterion (AICc) scores [76, 77]. The best-fitting model for an observer was the model that had the lowest AICc score. The ordering of model fit across observers was determined by the mean AICc score per model (i.e., the best-fitting model had the lowest average AICc score). Relative AICc scores are reported in text for the models best-fit by at least one observer and used for plotting purposes. Further details on the model fitting are provided in S1 Text. To confirm that the models are distinguishable for this experimental design, we performed a model recovery analysis. Using the MLE of the parameters, 10 data sets were simulated per observer per model. A successful model recovery indicates there is sufficient data to treat each participant as the replication unit (for more details on small-N designs, see [78]) and that different best-fitting models is less likely a result of measurement noise than model heterogeneity in the population [58, 59]. As such, the diversity in best-fitting model per participant is a meaningful result in the present study, reflecting idiosyncrasies in the confidence computation, and should be considered in conjunction with the group averages.

## Supporting information

S1 Text Supplementary information. 1) Model fitting: Derivation of the posterior for the Bayesian ideal observer; selecting the prior distribution parameters; computing the standard deviation of the marginal posterior distribution for the Scaled-Distance models; model-fitting procedure; and parameter recovery. 2) Additional results: preliminary logistic analysis to confirm that the quantity and quality manipulations affected confidence; qualitative comparison of the models; results of model variants; examining the Heuristic-model coefficients from the simulated datasets; and confidence agreement behaviour and model predictions.
(PDF)
