# Supplementary material for: Suprathreshold perceptual decisions constrain models of confidence
Source: PLoS Comput Biol. 2022 Jul 27;18(7):e1010318. doi: 10.1371/journal.pcbi.1010318 (PMC9359550; doi:10.1371/journal.pcbi.1010318)
Supplement: S1 Text — 1) Model fitting: Derivation of the posterior for the Bayesian ideal observer; selecting the prior distribution parameters; computing the standard deviation of the marginal posterior distribution for the Scaled-Distance models; model-fitting procedure; and parameter recovery. 2) Additional results: preliminary logistic analysis to confirm that the quantity and quality manipulations affected confidence; qualitative comparison of the models; results of model variants; examining the Heuristic-model coefficients from the simulated datasets; and confidence agreement behaviour and model predictions. (PDF) [file pcbi.1010318.s001.pdf]

S1 Text – Supplementary Information:
Suprathreshold perceptual decisions constrain models of confidence

Shannon M. Locke[a], Michael S. Landy[b,c], Pascal Mamassian[a]

a) Laboratoire des Systèmes Perceptifs, Département d'Études Cognitives, École Normale Supérieure, PSL University, CNRS, Paris, France
b) Department of Psychology, New York University, New York, New York, United States of America
c) Center for Neural Science, New York University, New York, New York, United States of America

Contents

1 Model fitting **2**

1.1 Derivation of the posterior for the Bayesian ideal observer 2

1.2 Selecting the prior distribution parameters 7

1.3 Computing the standard deviation of the marginal posterior distribution for the Scaled-Distance models 8

1.4 Model-fitting procedure 8

1.5 Parameter recovery 12

2 Additional results **12**

2.1 Preliminary logistic analysis to confirm that quantity and quality manipulations affected confidence 12

2.2 Qualitative comparison of the models 14

2.3 Results of model variants 16

2.4 Examining the Heuristic-model coefficients from the simulated datasets 17

2.5 Confidence agreement behaviour and model predictions 18

1 Model fitting

1.1 Derivation of the posterior for the Bayesian ideal observer

Here we show the derivation of the posterior distribution presented in the main paper. Note that for the sake of space, we are using $\mu = \mu_{cloud}$ for the mean of the dot-sampling distribution, $\tau = \tau_{cloud}$ the precision of this distribution, and $\tau_c = \tau_{comb}$ the combined precision.

Prior: The prior for the mean and precision of the dot-generation distribution can be defined as the following normal-Gamma distribution:

$$\begin{aligned} p(\mu, \tau) &= \mathcal{NG}(\mu, \tau | \mu_0, \kappa_0, \alpha_0, \beta_0) \\ &= \mathcal{N}(\mu | \mu_0, (\kappa_0 \tau)^{-1}) \mathcal{G}(\tau | \alpha_0, \beta_0) \\ &\propto \tau^{\frac{1}{2}} \exp\left(\frac{-\kappa_0 \tau}{2} (\mu - \mu_0)^2 \right) \times \tau^{\alpha_0 - 1} \exp(-\tau \beta_0). \end{aligned} \tag{S1}$$

The parameters μ_0 and κ_0 relate to the normal component, and α_0 and β_0 to the Gamma component.

Likelihood: The observer must consider two sources of uncertainty when choosing a likelihood function, the noisy draws of N dots from the dot-generation distribution and the measurement noise applied to each dot, σ^2_{dot} (precision: τ_{dot}). These two noise sources are additive, resulting in the combined precision per dot of

$$\tau_c = \frac{1}{\sigma^2_{cloud} + \sigma^2_{dot}} = \frac{1}{\tau^{-1} + \tau^{-1}_{dot}}. \tag{S2}$$

The observer must consider the information provided by all dot locations $\boldsymbol{X}$ (that are the horizontal component of physical dot locations $\boldsymbol{D}$ corrupted by measurement noise), resulting in the following likelihood function:

$$\begin{aligned} p(\boldsymbol{X} | \mu, \tau, \tau_{dot}) &= \prod_{i=1}^{N} p(x_i | \mu, \tau_c) \\ &= \prod_{i=1}^{N} \frac{1}{(2\pi)^{\frac{1}{2}}} \tau_c^{\frac{1}{2}} \exp\left(\frac{-\tau_c}{2} (x_i - \mu)^2 \right) \\ &\propto \tau_c^{\frac{N}{2}} \exp\left(\frac{-\tau_c}{2} \sum_{i=1}^{N} (x_i - \mu)^2 \right), \end{aligned} \tag{S3}$$

where τ_c is defined in Eq. S2 and we assume the observer has accurate knowledge of σ^2_{dot}. Note that we have dropped the constant terms from the equation for simplicity as they can be applied by re-scaling

the posterior.

Posterior: As we have used a conjugate prior, the posterior is also a normal gamma of the form $\mathcal{NG}(\mu, \tau|\mu_p, \kappa_p, \alpha_p, \beta_p, \tau_{dot})$. However, to see this clearly, we decompose the posterior into three parts (A, B, and C).

$$\begin{aligned} p(\mu, \tau|\boldsymbol{X}, \tau_{dot}) &\propto \text{prior} \times \text{likelihood} \\ &\propto \tau^{\frac{1}{2}} \exp\left(\frac{-\kappa_0 \tau}{2}(\mu - \mu_0)^2\right) \tau^{\alpha_0 - 1} \exp(-\tau\beta_0) \tau_c^{\frac{N}{2}} \exp\left(\frac{-\tau_c}{2}\sum_{i=1}^{N}(x_i - \mu)^2\right) \\ &\propto A \times B \times C. \end{aligned} \tag{S4}$$

Part A is the non-exponent term of the normal component,

$$A = \tau^{\frac{1}{2}}. \tag{S5}$$

Part B is the non-exponentiated term of the Gamma component, which can be rearranged as follows

$$\begin{aligned} B &= \tau^{\alpha_0 - 1} \tau_c^{\frac{N}{2}} \\ &= \tau^{\alpha_0 - 1} \left(\frac{\tau}{\tau_{dot}^{-1}\tau + 1}\right)^{\frac{N}{2}} \\ &= \tau^{\alpha_0 + N/2 - 1} \exp\left(\frac{-N}{2}\log(\tau_{dot}^{-1}\tau + 1)\right). \end{aligned} \tag{S6}$$

The non-exponent part of B will be the non-exponent term of the Gamma component, and the remainder will be placed in Part C:

$$C = \exp\left(\frac{-\kappa_0 \tau}{2}(\mu - \mu_0)^2\right) \exp(-\tau\beta_0) \exp\left(\frac{-\tau_c}{2}\sum_{i=1}^{N}(x_i - \mu)^2\right). \tag{S7}$$

Thus, Part B becomes

$$B' = \tau^{\alpha_0 + N/2 - 1} = \tau^{\alpha_p - 1}, \tag{S8}$$

with

$$\alpha_p = \alpha_0 + N/2, \tag{S9}$$

and Part C, which now aggregates all the exponent terms, is

$$
\begin{aligned}
C' &= \exp\left(\frac{-\kappa_0\tau}{2}(\mu-\mu_0)^2\right)\exp(-\tau\beta_0)\exp\left(\frac{-\tau_c}{2}\sum_{i=1}^{N}(x_i-\mu)^2\right)\exp\left(\frac{-N}{2}\log(\tau_{dot}^{-1}\tau+1)\right)\\
&= \exp\left(-\frac{1}{2}\left[\kappa_0\tau(\mu-\mu_0)^2+\tau_c\sum_{i=1}^{N}(x_i-\mu)^2\right]-\tau\beta_0-\frac{N}{2}\log(\tau_{dot}^{-1}\tau+1)\right)\\
&= \exp\left(-\frac{1}{2}D-\tau\beta_0-\frac{N}{2}\log(\tau_{dot}^{-1}\tau+1)\right),
\end{aligned}
\tag{S10}
$$

which will contribute to both the normal and the Gamma components. Part D of this equation can be simplified, by extracting Part E as follows

$$
\begin{aligned}
D &= \kappa_0\tau(\mu-\mu_0)^2+\tau_c\sum_{i=1}^{N}(x_i-\mu)^2\\
&= \kappa_0\tau(\mu-\mu_0)^2+N\tau_c(\mu-\bar{x})^2+\tau_c\sum_{i=1}^{N}(x_i-\bar{x})^2\\
&= E+\tau_c\sum_{i=1}^{N}(x_i-\bar{x})^2,
\end{aligned}
\tag{S11}
$$

given that

$$
\begin{aligned}
\sum_{i=1}^{N}(x_i-\mu)^2 &= \sum_{i=1}^{N}[(x_i-\mu-\bar{x}+\bar{x})]^2\\
&= \sum_{i=1}^{N}(\bar{x}-\mu)^2+\sum_{i=1}^{N}(x_i-\bar{x})^2-2\sum_{i=1}^{N}(x_i-\bar{x})(\mu-\bar{x})\\
&= N(\bar{x}-\mu)^2+\sum_{i=1}^{N}(x_i-\bar{x})^2,
\end{aligned}
\tag{S12}
$$

and 34

$$
\sum_{i=1}^{N}(x_i-\bar{x})(\mu-\bar{x}) = (\mu-\bar{x})\left(\sum_{i=1}^{N}x_i-N\bar{x}\right) = (\mu-\bar{x})(N\bar{x}-N\bar{x}) = 0.
\tag{S13}
$$

Afterwards, completing the square[1] and rearranging, we get

$$
\begin{aligned}
E &= \kappa_0\tau(\mu-\mu_0)^2 + N\tau_c(\mu-\bar{x})^2 \\
&= \kappa_0\tau(\mu^2-2\mu\mu_0+\mu_0^2) + N\tau_c(\mu^2-2\mu\bar{x}+\bar{x}^2) \\
&= \mu^2(\kappa_0\tau+N\tau_c) - 2\mu(\kappa_0\tau\mu_0+N\tau_c\bar{x}) + \kappa_0\tau\mu_0^2 + N\tau_c\bar{x}^2 \\
&= (\kappa_0\tau+N\tau_c)(\mu-\mu_p)^2 + \kappa_0\tau\mu_0^2 + N\tau_c\bar{x}^2 - \frac{(\kappa_0\tau\mu_0+N\tau_c\bar{x})^2}{\kappa_0\tau+N\tau_c} \\
&= (\kappa_0\tau+N\tau_c)(\mu-\mu_p)^2 + \frac{\kappa_0\tau\mu_0^2(\kappa_0\tau+N\tau_c) + N\tau_c\bar{x}^2(\kappa_0\tau+N\tau_c) - (\kappa_0\tau\mu_0+N\tau_c\bar{x})^2}{\kappa_0\tau+N\tau_c} \\
&= (\kappa_0\tau+N\tau_c)(\mu-\mu_p)^2 + \frac{\kappa_0 N\tau\tau_c(\bar{x}-\mu_0)^2}{\kappa_0\tau+N\tau_c},
\end{aligned}
\tag{S14}
$$

where

$$
\mu_p = \frac{\kappa_0\tau\mu_0+N\tau_c\bar{x}}{\kappa_0\tau+N\tau_c}. \tag{S15}
$$

Thus, Part D can be written as

$$
D = (\kappa_0\tau+N\tau_c)(\mu-\mu_p)^2 + \frac{\kappa_0 N\tau\tau_c(\bar{x}-\mu_0)^2}{\kappa_0\tau+N\tau_c} + \tau_c\sum_{i=1}^{N}(x_i-\bar{x})^2, \tag{S16}
$$

and we can now express Part C' in terms of two components, C_1 and C_2,

$$
\begin{aligned}
C' &= \exp\Big(-\frac{1}{2}D - \tau\beta_0 - \frac{N}{2}\log(\tau_{dot}^{-1}\tau+1)\Big) \\
&= \exp\Big(-\frac{1}{2}(\kappa_0\tau+N\tau_c)(\mu-\mu_p)^2\Big)\times \\
&\quad \exp\Big(-\tau\beta_0 - \frac{\kappa_0 N\tau\tau_c(\bar{x}-\mu_0)^2}{2(\kappa_0\tau+N\tau_c)} - \frac{\tau_c}{2}\sum_{i=1}^{N}(x_i-\bar{x})^2 - \frac{N}{2}\log(\tau_{dot}^{-1}\tau+1)\Big) \\
&= C_1\times C_2,
\end{aligned}
\tag{S17}
$$

[1]Completing the square: $ax^2+bx+c=a(x+d)^2+e$, where $d=b/2a$ and $e=c-b^2/4a$.

which contribute to the normal component and the Gamma component respectively. To see this, we must arrange Part C_1 as follows

$$\begin{aligned} C_1 &= \exp\Bigg(-\frac{1}{2}(\kappa_0\tau + N\tau_c)(\mu - \mu_p)^2 \Bigg) \\ &= \exp\Bigg(\frac{-\tau(\kappa_0\tau + N\tau_c)}{2\tau}(\mu - \mu_p)^2 \Bigg) \\ &= \exp\Bigg(\frac{-\kappa_p\tau}{2}(\mu - \mu_p)^2 \Bigg), \end{aligned} \tag{S18}$$

where

$$\kappa_p = \frac{\kappa_0\tau + N\tau_c}{\tau}. \tag{S19}$$

Similarly, for Part C_2,

$$\begin{aligned} C_2 &= \exp\Bigg(-\tau\beta_0 - \frac{\kappa_0 N\tau\tau_c(\bar{x} - \mu_0)^2}{2(\kappa_0\tau + N\tau_c)} - \frac{\tau_c}{2}\sum_{i=1}^{N}(x_i - \bar{x})^2 - \frac{N}{2}\log(\tau_{dot}^{-1}\tau + 1) \Bigg) \\ &= \exp\Bigg(-\tau\Bigg[\beta_0 + \frac{\kappa_0 N\tau\tau_c(\bar{x} - \mu_0)^2}{2\tau(\kappa_0\tau + N\tau_c)} + \frac{\tau_c}{2\tau}\sum_{i=1}^{N}(x_i - \bar{x})^2 + \frac{N}{2\tau}\log(\tau_{dot}^{-1}\tau + 1)\Bigg] \Bigg) \\ &= \exp\Bigg(-\tau\Bigg[\beta_0 + \frac{N}{2\tau}\Bigg(\frac{\kappa_0\tau\tau_c(\bar{x} - \mu_0)^2}{\kappa_0\tau + N\tau_c} + \tau_c s^2 + \log(\tau_{dot}^{-1}\tau + 1)\Bigg)\Bigg] \Bigg) \\ &= \exp(-\tau\beta_p), \end{aligned} \tag{S20}$$

where

$$\beta_p = \beta_0 + \frac{N}{2\tau}\Bigg(\frac{\kappa_0\tau\tau_c(\bar{x} - \mu_0)^2}{\kappa_0\tau + N\tau_c} + \tau_c s^2 + \log(\tau_{dot}^{-1}\tau + 1)\Bigg), \tag{S21}$$

and s^2 is the sample variance

$$s^2 = \frac{1}{N}\sum_{i=1}^{N}(x_i - \bar{x})^2. \tag{S22}$$

To see how this is a normal-Gamma we reassemble

$$\begin{aligned} p(\mu, \tau | \boldsymbol{X}, \tau_{dot}) &\propto AC_1 \times B'C_2 \\ &\propto \tau^{\frac{1}{2}} \exp\Bigg(\frac{-\kappa_p\tau}{2}(\mu - \mu_p)^2 \Bigg) \times \tau^{\alpha_p - 1}\exp(-\tau\beta_p) \\ &\propto \mathcal{NG}(\mu, \tau | \mu_p, \kappa_p, \alpha_p, \beta_p). \end{aligned} \tag{S23}$$

Validation: We can check this derivation against the simpler case of $p(\mu, \tau|\boldsymbol{D})$, which has been documented previously in the literature [1]. The important distinction between these derivations is

that there is no additive noise per dot in the likelihood for $p(\mu, \tau|\boldsymbol{D})$, with the consequence that $\tau_c = \tau$. In this case, the parameters of the posterior simplify as follows:

$$\mu_p = \frac{\kappa_0 \tau \mu_0 + N\tau_c \bar{x}}{\kappa_0 \tau + N\tau_c} = \frac{\kappa_0 \mu_0 + N\bar{x}}{\kappa_0 + N}, \tag{S24}$$

$$\kappa_p = \frac{\kappa_0 \tau + N\tau_c}{\tau} = \kappa_0 + N, \tag{S25}$$

$$\alpha_p = \alpha_0 + N/2, \tag{S26}$$

and

$$\begin{aligned} \beta_p &= \beta_0 + \frac{N}{2\tau}\left(\frac{\kappa_0 \tau \tau_c (\bar{x} - \mu_0)^2}{\kappa_0 \tau + N\tau_c} + \tau_c s^2 + \log(\tau_{dot}^{-1}\tau + 1)\right) \\ &= \beta_0 + \frac{\kappa_0 N(\bar{x} - \mu_0)^2}{2(\kappa_0 + N)} + \frac{Ns^2}{2}. \end{aligned} \tag{S27}$$

These parameter equations match those reported in the derivation of $p(\mu, \tau|\boldsymbol{D})$ in [1].

1.2 Selecting the prior distribution parameters

We adjusted the parameters of the normal-Gamma prior ($\mu_0, \kappa_0, \alpha_0$, and β_0) to the true stimulus statistics of the dots mean μ and precision τ in the experiment. The possible centre locations of the dots were -4, -2, -1, 0, 1, 2, or 4 deg, and the possible standard deviations of the dots spreads were 1.5, 2, or 2.5 deg. Therefore, the statistics of the mean and precision were $E[\mu] = 0$, $\mathrm{Var}[\mu] = 7$, $E[\tau] = 0.2848$, and $\mathrm{Var}[\tau] = 0.0211$.

We matched the marginal distributions of the mean and precision variables to these statistics. The marginal distribution of the precision τ variable is the Gamma distribution with parameters (α_0, β_0). The mean and variance of this distribution are

$$\mathrm{E}[\tau] = \frac{\alpha_0}{\beta_0}, \tag{S28}$$

$$\mathrm{Var}[\tau] = \frac{\alpha_0}{\beta_0^2}. \tag{S29}$$

The marginal distribution of the mean μ variable is the non-standardized Student's t-distribution with parameters $(\nu_0, \mu_0, \sigma_0^2) = (2\alpha_0, \mu_0, \beta_0/(\kappa_0\alpha_0))$. The mean and variance of this distribution are

$$\mathrm{E}[\mu] = \mu_0, \tag{S30}$$

$$\mathrm{Var}[\mu] = \frac{\sigma_0^2 \nu_0}{\nu_0 - 2} = \frac{\beta_0}{\kappa_0 \alpha_0} \frac{2\alpha_0}{2\alpha_0 - 2} = \frac{\beta_0}{\kappa_0(\alpha_0 - 1)}. \tag{S31}$$

The match of these marginal statistics results in the following prior distribution parameters

$$\mu_0 = \mathrm{E}[\mu] = 0, \tag{S32}$$

$$\kappa_0 = \frac{\mathrm{E}[\tau]}{\mathrm{Var}[\mu](\mathrm{E}[\tau]^2 - \mathrm{Var}[\tau])} = 0.68, \tag{S33}$$

$$\alpha_0 = \frac{\mathrm{E}[\tau]^2}{\mathrm{Var}[\tau]} = 3.84, \tag{S34}$$

and

$$\beta_0 = \frac{\mathrm{E}[\tau]}{\mathrm{Var}[\tau]} = 13.48. \tag{S35}$$

1.3 Computing the standard deviation of the marginal posterior distribution for the Scaled-Distance models

The Scaled-Distance models normalised the distance-From-Criterion (DFC) of the posterior mode, $\hat{\mu}$, by the standard deviation of the marginal posterior distribution, $\hat{\sigma}$, for each interval. We calculated the scaled DFC in the model fitting procedure using an inverse cumulative normal function approximation.

$$\mathrm{DFC}_{\mathrm{scaled}} = |\frac{\hat{\mu} - k_1}{\hat{\sigma}} + \epsilon| \approx |\Phi_{\mathrm{inv}}\big(p(C = R|\boldsymbol{X}, k_1)\big) + \epsilon|. \tag{S36}$$

Note the additive confidence noise, $\epsilon \sim N(0, \sigma^2_{\mathrm{conf}})$.

1.4 Model-fitting procedure

Type 1 models. Type 1 models were fit first, with σ_{dot}, k_1, and λ as free parameters. Models were fit on a per-participant basis with custom MATLAB scripts that calculated the likelihood of the model for specific parameter combinations in a dense grid of parameter values (σ_{dot}: 0.1 to 2 deg in 50 log-spaced steps; k_1: -1 to 1 deg in 20 steps; λ: 0.01 to 0.1 in 9 steps). Choice probabilities for each parameter combination were estimated by simulation. For each trial, 1000 observations were simulated, with choice probabilities estimated by computing the proportion of rightward choices made by the simulated observer. This involved constructing the prior, likelihood, and posterior distributions shown in Figure 6A of the main paper (grid resolution: μ: -10 to 10 in 200 steps; τ: 0.01 to 1.00 in 100 steps). After marginalising the posterior distribution across the τ dimension, the choice probabilities could be calculated using k_1 and λ. Because this was a computationally expensive simulation, the simulated

noisy observations and construction of the prior, likelihood, and posterior grids was done only once per level of σ_{dot} (for each trial and for each participant). Thus differences in choice probabilities from k_1 and λ were calculated using the same 1000 noisy simulations of the trial. Not taking this approach would have increased the time to fit a single Type 1 model for an observer from hours to days or weeks. Choice probabilities were then put into a Bernoulli model of binary choice behaviour with the participant's actual responses to calculate the likelihood of that specific parameter combination. Finally, the best-fitting parameters were extracted from the 3D parameter grid by finding combination that gave the largest the maximum likelihood value. Averages of the best-fitting parameter values are given in Table A.

Type 2 models. The Type 2 models were fit using the same simulation-based approach combined with Bayesian Adaptive Direct Search (BADS)[2] to find the best-fitting combination of parameters. The number of parameters in the Type 2 model fits depended on the model. The Ideal-Confidence-Observer model had no free parameters, the Heuristic model had 4 free parameters (β_1, β_2, σ_{conf}, k_2), and the remaining models had two free parameters (ν or σ_{conf}, k_2). The Type 1 parameters were fixed at their best-fitting values on a per-participant basis and the Type 1 model was matched to the Type 2 model in terms of whether the flat- or centred-prior variant was used because the location of a biased k_1 can differ between the variants.

The first step in Type 2 model fits was simulating the observer. For each participant, we simulated 3000 noisy observations of each trial, and computed the marginalised posterior distribution using the same μ-τ grid spacing as in the Type 1 model-fitting procedure. For each simulation, we computed $p(C = R|X, k_1)$ of Eq. 12 as well as the mode of the posterior distribution according to the prior variants of the Type 1 models. Each simulation was then coded as favouring a leftward or rightward Type 1 response. We sampled with replacement from the simulations that matched the Type 1 response given by the participant to ensure the simulated observer matched the actual observer in Type 1 choice behaviour. If all simulations were choice-consistent for a given trial we did not perform this sampling step. If no samples were choice-consistent, the trial was flagged as a likely lapse for later processing. Due to the mostly low difficulty of the task, the majority of samples usually favoured the same response and it was rare for the sampling to be drawn from a small number of simulations. This simulation procedure was performed only once per trial and observer and used for the fitting of all Type 2 models.

In the second step, we fit each of the Type 2 models with the set of choice-consistent simulations

using BADS. We considered the relative evidence for the Type 1 response given by the observer in each interval, according to the Type 2 model. Likely lapses were given the lowest confidence value possible with a tiny amount of jitter so the confidence report for comparing two lapses is entirely random. Thus, we were able to compute the choice probabilities for selecting “Interval 1” and “Interval 2” from the simulated observer. These were compared with the actual confidence forced-choice report of the participant in a Bernoulli model of binary choice behaviour. As choice probabilities were computed by simulation, we used the “uncertainty handling” option in the BADS package and drew 100 final samples to compute the estimate of the negative log-likelihood value of the model for the model comparison. The lower bound, upper bound, plausible lower bound, plausible upper bound, and starting location settings for each model can be found in the *fitType2.m* file available at `https://osf.io/k2nhq/`. Averages of the best-fitting parameter values per Type 2 model are given in Table A.

Table A: Summary of best-fitting parameters per model. Type 1 model fits were near identical, so parameter values were averaged across the four models for each observer before computing the parameter averages across observers. Type 2 models are detailed individually. Where relevant, the parameters from fits of the flat- and centred-prior variants are both reported.

**Model**	**Parameter**	**Mean±SEM**
All Type 1 (averaged)	σ_{dot}	0.97° ± 0.04°
	k_1	0.01° ± 0.03°
	λ	0.02±0.002
Ideal Confidence Observer	none	none
Basic Probability	ν_{conf}	6.30±1.24; 4.91±0.90
	k_2	0.001±0.001; 0.001±0.001
Probability Difference	σ_{conf}	0.19±0.03; 0.21±0.03
	k_2	0.02±0.01; 0.02±0.01
Log Probability Ratio	σ_{conf}	2.29±0.29; 2.15±0.28
	k_2	0.11±0.16; 0.09±0.14
Unscaled Distance	σ_{conf}	2.08° ± 0.18°; 1.42° ± 0.12°
	k_2	0.04° ± 0.11°; 0.03° ± 0.08°
Scaled Distance	σ_{conf}	0.98±0.11 sd; 0.94±0.11 sd
	k_2	0.05±0.07 sd; 0.04±0.06 sd
Heuristic	σ_{conf}	1.12±0.17
	k_2	0.13±0.08
	β_1 (quantity)	0.68±0.10
	β_2 (quality)	0.38±0.09

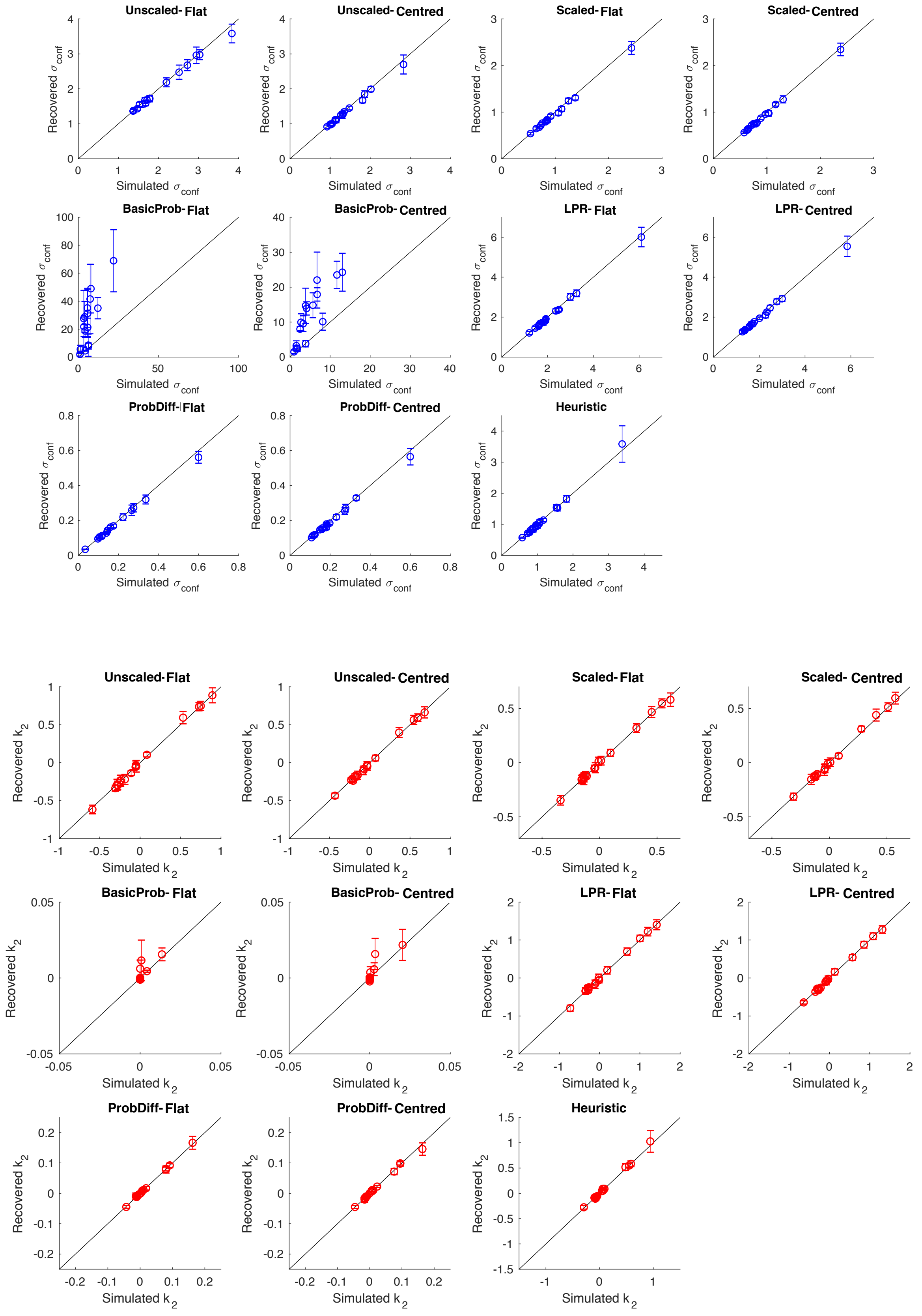

Figure A: Parameter recovery results for σ_{conf} (blue) and k_2 (red). Model and prior variant indicated by title. Data points are the mean values and error bars are ±1 SD calculated from the fits of the 10 simulated data sets per observer per model.

1.5 Parameter recovery

To confirm that the model-fitting procedure was behaving correctly, we assessed whether refit models in the model-recovery analysis recovered the simulated parameters. Figure A shows that the confidence noise and bias terms were recovered well in almost all instances. The exception was the confidence-noise parameter for the Basic-Probability model, where the magnitude of the noise was often overestimated. We investigated this further and found that the Beta noise distribution was little changed for different confidence noise values for extreme probability values (i.e., close to 0 or 1). Thus, the design of the experiment likely affected the ability to fit this model.

2 Additional results

2.1 Preliminary logistic analysis to confirm that quantity and quality manipulations affected confidence

We examined whether the evidence quantity and quality manipulations affected confidence judgements. Observers could have ignored the sensory-uncertainty manipulations, and simply reported whichever interval had the more extreme leftward or rightward stimulus as more confident (the *Basic* model). Alternatively, the observer may modulate their confidence by information quantity and/or quality: increasing confidence if there were 5 dots or smaller spread, and decreasing it for 2 dots or larger spread. We considered these four possibilities (Basic, only quantity, only quality, and both) in a nested model comparison. It is reasonable to suspect that the observer inferred the mean and spread from the displayed dots, so we repeated the analysis for both the true mean and spread of the generating distribution and the empirical mean and spread values according to the dots displayed. If decision correctness is examined, observer reports better match the empirical mean of the dots (i.e., the centroid) as opposed to the mean of generating distribution (Figure B(A)).

All models for the preliminary analysis were a logistic function of the probability of choosing Interval 2 as more confident,

$$p(\text{choose } I_2) = \lambda + (1 - 2\lambda)Logit(\theta), \tag{S37}$$

with the predicted probabilities scaled according to the lapse rate, λ ($0.01 \leq \lambda \leq 0.15$). Here lapse rate is the rate of unintentionally giving the opposite decision than desired. The *Full* model had three predictors related to the distance of the stimuli from the screen centre, the number of dots, and the

dot spreads in each interval:

$$\theta_{\text{Full}} = \beta_0 + \beta_1 \Delta|\mu| + \beta_2 \Delta N_{\text{dots}} + \beta_3 \Delta \frac{1}{\sigma_{\text{cloud}}}. \quad \text{(S38)}$$

The constant term, β_0, is the confidence bias for choosing Interval 2 and was included in all models. The relative distance of the stimuli was parameterised as the relative absolute distance of the dot centroids from the screen centre, $\Delta|\mu|$, and was also included in all models. The relative number of dots, ΔN_{dots}, and the inverse of the spreads, $\Delta 1/\sigma_{\text{cloud}}$, were optional predictors in the nested models. The *Basic* model did not consider the evidence quantity or quality manipulations when computing confidence,

$$\theta_{\text{Basic}} = \beta_0 + \beta_1 \Delta|\mu|. \quad \text{(S39)}$$

Compared to the Basic model, the +*Quantity* model that included the dot-number predictor (β_2) and the +*Quality* model that included the dot-spread predictor (β_3) provided a better fit to the data, with the +Quantity model fitting better than the +Quality model (Figure B(B)). This was true both if the generating distribution spread was used or if the empirical spread was used (i.e., based on the displayed dots). The empirical version did fit slightly better overall.

The models were fit with custom MATLAB scripts that calculated the maximum-likelihood estimates of the parameters using gradient decent. Corrected Akaike information criterion (AICc) scores [3, 4] were computed for model comparison from the log-likelihood values. AICc scores explicitly

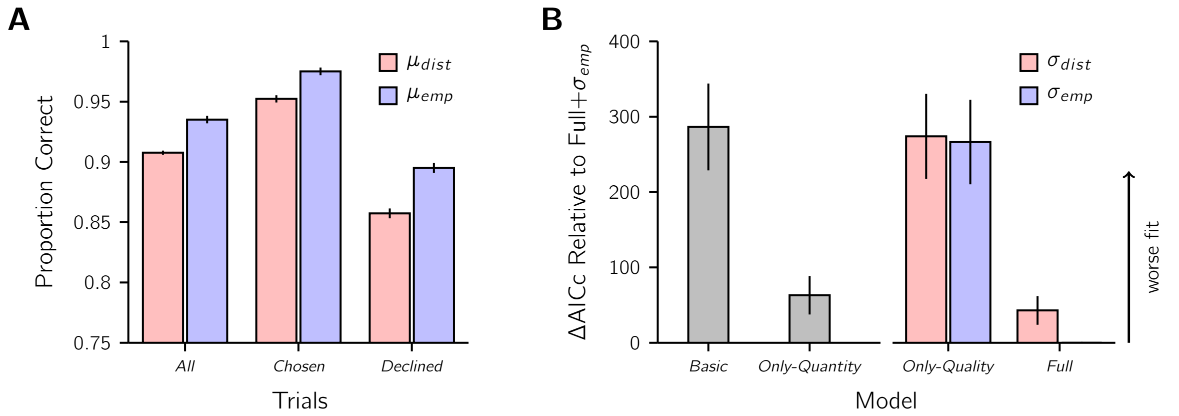

Figure B: Preliminary results. A) Type 1 judgements. Task performance when decision correctness is scored against the true mean of the generating distribution (pink) versus the empirical mean of the dot cloud (purple). B) Type 2 Logistic regression results. Model fit is compared against the winning model: the Full model with empirical spread. Grey: models without the quality predictor. Pink: models with a quality predictor based on the true generating distribution spread. Purple: quality predictor based on the empirical spread. All error bars: ±SEM.

penalise the number of parameters in a model and correct for sample size.

We also repeated this nested analysis using a restricted dataset of only trial pairs where both Type 1 judgements were correct. The purpose of this analysis was to reduce the probability that lapse discrimination responses were included, because the logistic regression did not consider Type 1 performance in the confidence report. The pattern of model fits, however, did not change.

2.2 Qualitative comparison of the models

To qualitatively evaluate the models, we used the simulations from the model and parameter recovery analyses. Figure C shows the confidence choice probabilities as a function of the three stimulus manipulations (number of dots, dot-cloud SD and distance of the dot cloud from the centre). There are three notable differences between the models from this perspective. First, the Unscaled-Distance model is largely insensitive to the quality and quantity manipulations of the sensory uncertainty as it simply measures the distance between the peak of the posterior and the centrally-located decision criterion (there will be no effect in the flat-prior variant, as depicted, and a small effect in the centred-prior variant, which is not shown). As such, the pattern of confidence choice-probabilities is symmetric around the vertical midline, with nearly equal confidence probabilities for the columns of different binned inverse dot-spreads and the same or different dot-number panels. The second observation is that the Ideal model has a greater colour saturation because the lack of confidence noise leads to more extreme confidence choice-probability values. The probabilities near the gold confidence-indifference contours are closer to chance levels due to the presence of Type 1 noise only. Lastly, the Heuristic model is better able to capture the bias in confidence for comparisons of dot-clouds that differ in the number of dots (right panel). This is due to this model's flexibility to capture the extreme over-weighting of the quantity of dots prevalent in the sub-group of observers best fit by the Heuristic model. All the other models, except the flat-prior variant of the Unscaled-Distance model, are constrained by the actual sensory uncertainty present in the stimulus.

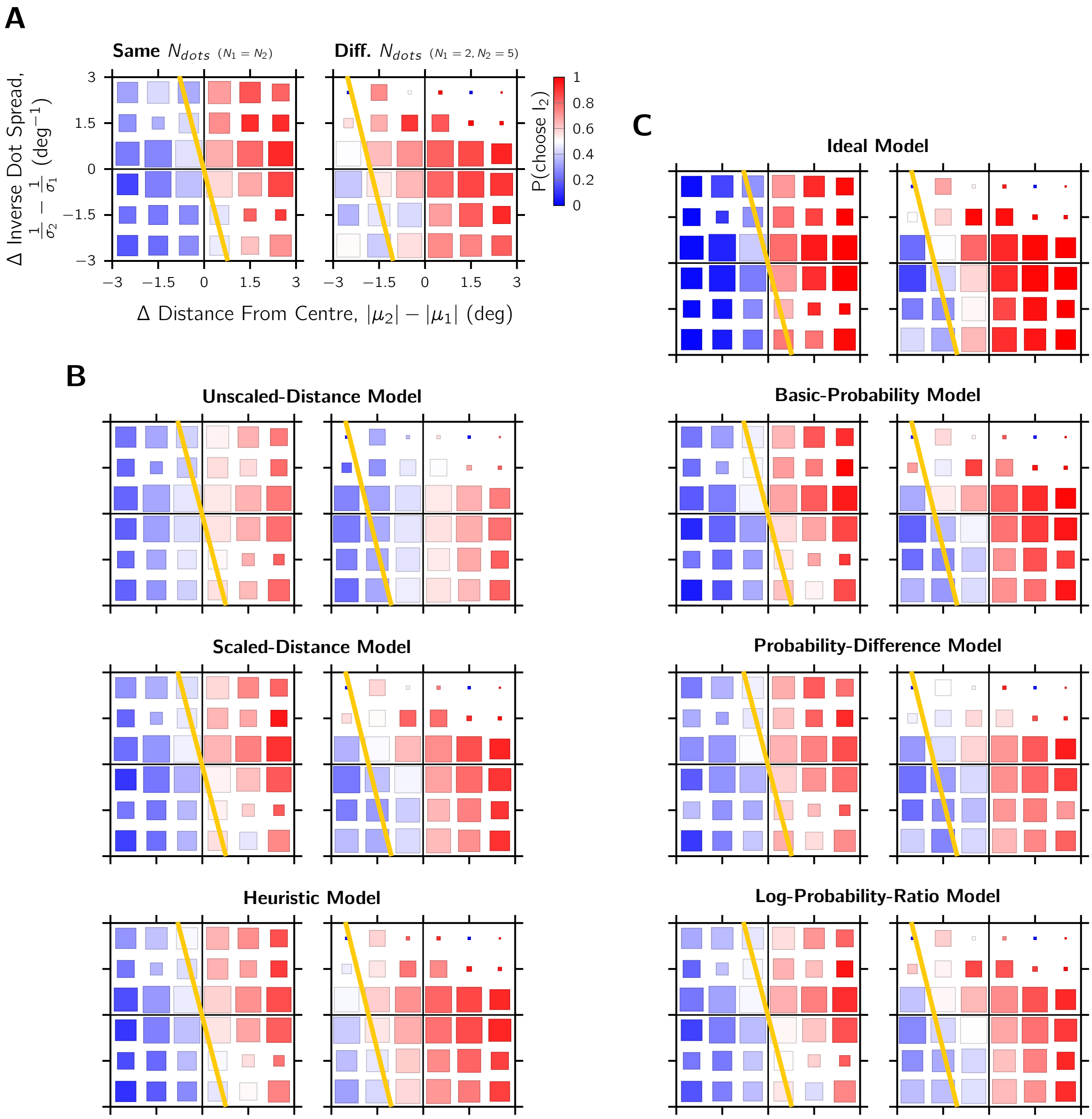

Figure C: Qualitative comparison of the models. A) A replication of Figure 3B-C. Raw confidence choices, sorted by stimulus properties across the two intervals of a confidence pair. The colour code represents the proportion of "Interval 2" as more confident choices averaged across observers. Confidence choices are plotted as a function of the difference in distance from centre across intervals and difference in inverse dot spread. The distance from centre and dot spread were calculated using the empirical mean and SD of the dots displayed, and binned in the range $\pm 3°$ for plotting. Gold line: the confidence-indifference contour, where the observer is equally likely to report Interval 1 or 2, calculated from the Full model in the nested logistic regression analysis. B) The confidence choice data based on the simulations of the three DFC Evidence-Strength models. There were 100 simulated datasets per model per observer based on the observer's best-fitting parameters for that model type. Where relevant, only the flat-prior variants are shown due to a high similarity with the centred-prior variants. C) The same as in B for the four Probability-metric models.

2.3 Results of model variants

The base model assumed that the likelihood function was calculated from the horizontal locations of the noisy dot measurements. In the centred-prior variant, the prior distribution was a normal-Gamma distribution matched to the stimulus statistics. These modelling choices were investigated by checking whether the main experimental results were robust to changes in this general base model.

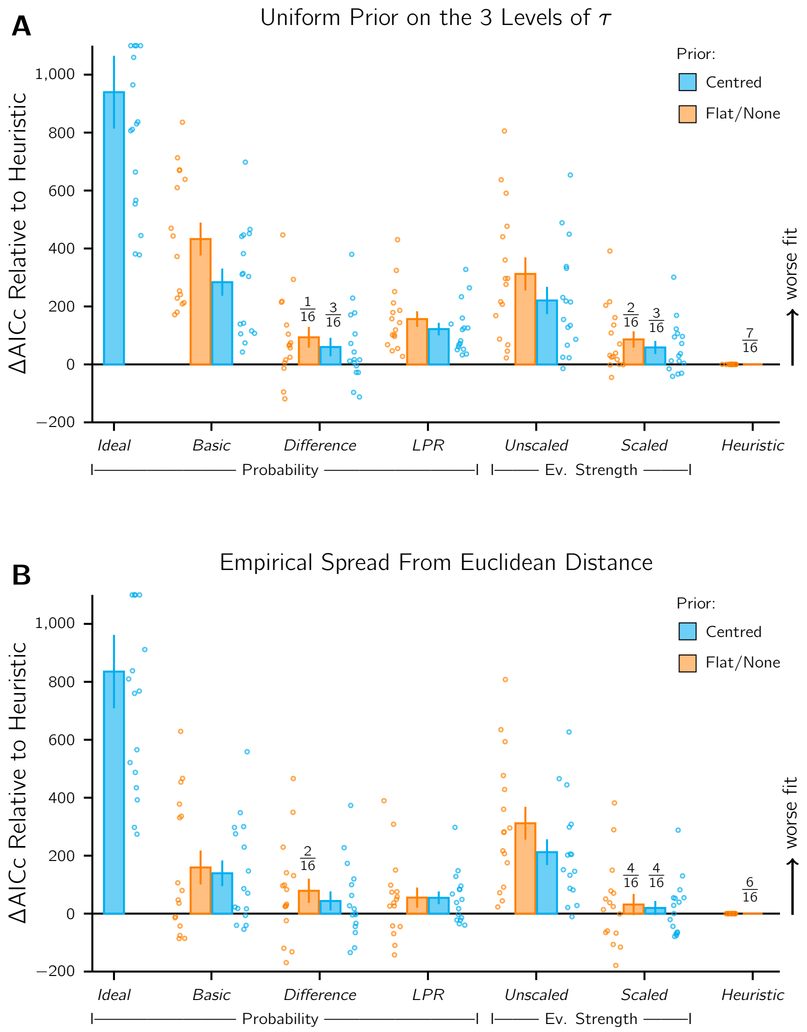

Figure D: Type 2 model-comparison results when the form of the general base model was changed. A) Models with a uniform prior on τ instead of the normal-Gamma prior. We assume here that the observer has perfect knowledge of the three levels of dot spread and implements a prior that gives equal weight to each of the three corresponding τ values. Bars: average relative AICc score. Markers: Individual participant results. Colour: flat-prior (orange) or centred-prior (blue) variant. Heuristic model has no prior. B) Models with the empirical spread computed from Euclidean distance instead of horizontal distance. Both the horizontal and vertical position of the dot are used in the standard deviation calculation. Error bars: ±SEM.

Uniform prior on the three precision levels. In this analysis, we changed the joint mean-precision prior of the centred-prior to reflect that the observer had perfect knowledge of the three levels of distribution spread used in the experiment. On an individual trial, the observer considered the prior probability of the precision as uniform across these three levels. The prior probability of the mean was matched to the stimulus statistics, $N(0, 7)$, for the centred-prior variant. The winning model remained the Heuristic model, with the number best fit for this model unchanged (Figure D(A)). However, the number of observers best-fit by the Probability-Difference model increased by 2, and the number of observers best-fit with a posterior variant doubled from 3 to 6. Note that the flat-prior variant results are unchanged in this analysis, so changes of the best-fitting model were a result of the centred-prior variants fitting better or worse.

Empirical spread from Euclidean distance. Here we included the vertical spread of the dots. The vertical position of the generating distribution mean was always the half-height of the screen. Yet, the distance of the dots from this half-height reference also give an indication of the true spread of the circular-symmetric Gaussian generating distribution. The Heuristic model remained the overall winner of the model comparison (Figure D(B)). However, now the Scaled-Distance model has the slightly more observers best fit compared to the Heuristic model. We do not consider this a significant departure from the results reported in the main article.

2.4 Examining the Heuristic-model coefficients from the simulated datasets

To better understand the relationship between the best-fitting coefficients from the Heuristic model and the best-fitting model for each observer (Figure E(A)), we examined the fits of the model simulations from the model-recovery analysis. In particular, there appeared to be some clustering of coefficient values based on the model type for the non-heuristic models. The model simulations, based on all observers, confirmed this clustering (Figure E(B)). It also showed a large spread in the coefficients for the Heuristic model simulations, consistent with the fits to the human data. A similar spread was observed for the Ideal-Confidence-Observer model simulations (Figure E(C)), and a small spread for the Basic-Probability models simulations. The simulated Unscaled-Distance model coefficients are near 0, consistent with these models having little to no influence of information quantity or quality on confidence, for the flat- and centred-prior variants respectively.

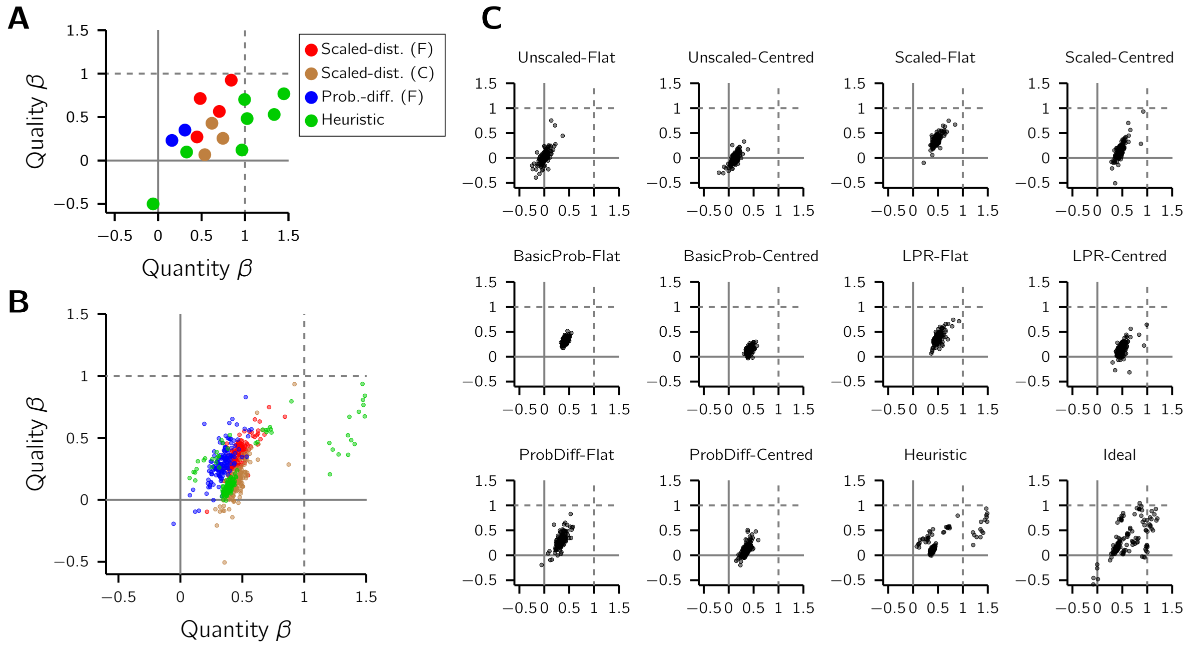

Figure E: Comparing the Heuristic-model fit for the behaviour of observers against the Heuristic-model fit for the model simulations. A) Replotting of observer fits from Figure 5D. Each datapoint is a single observer, coloured according to the best-fitting model (note change in colour scheme). Prior variants are flat (F) or centred (C). B) Same as in A for the simulations of the selected models. Each datapoint is the fit from a single model simulation (10 simulations per observer; best-fitting parameters used). C) The best-fitting Heuristic-model coefficients for each of the models.

2.5 Confidence agreement behaviour and model predictions

The confidence agreement behaviour of all observers, and their corresponding model predictions according to their best-fitting parameters are shown in Figure F. The pattern of results reveals the tendency of the observer to be more consistent than their best-fitting model would predict.

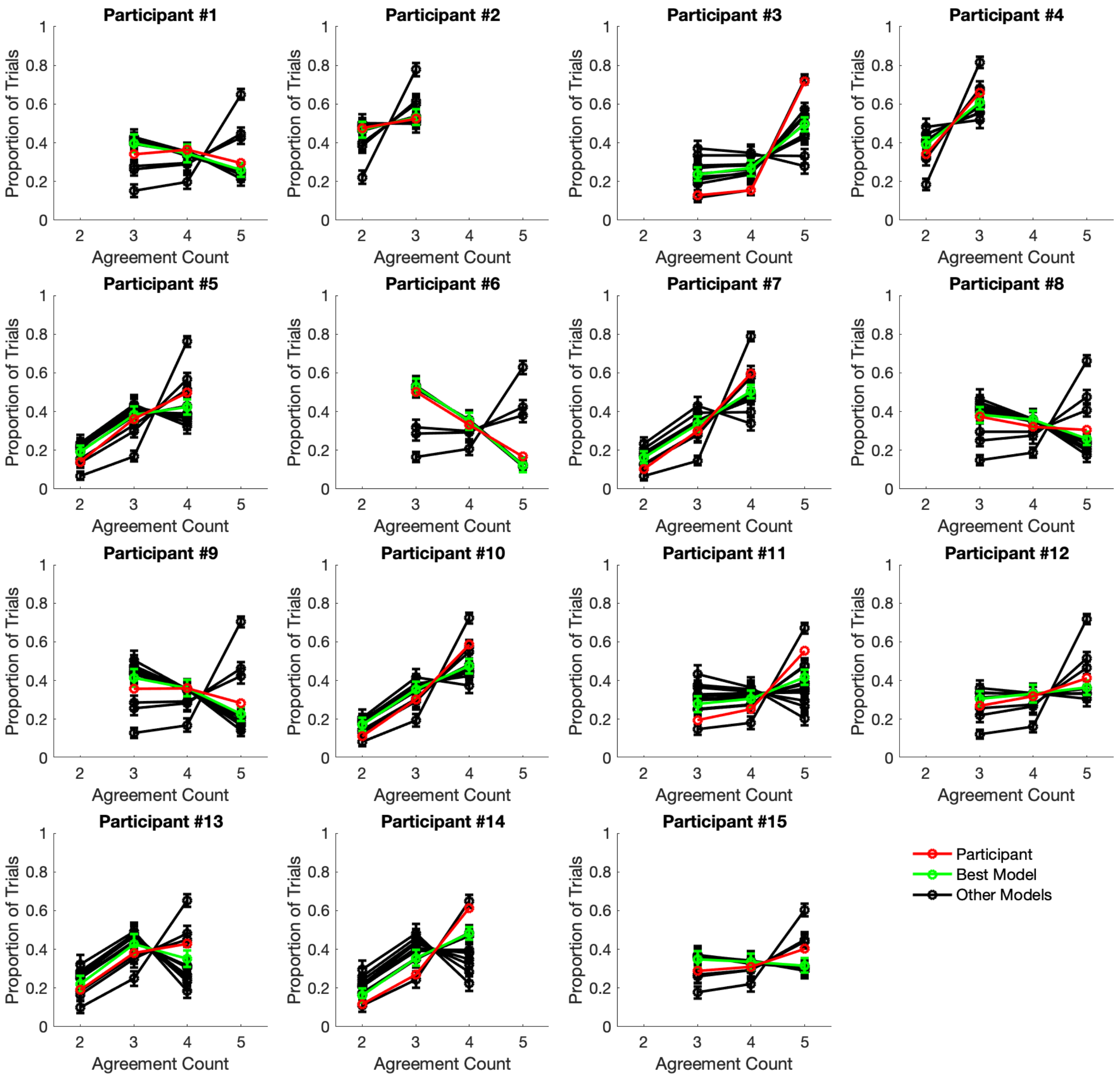

Figure F: The 5-pass confidence agreement of all observers with the predicted confidence agreement of each model. Model predictions were calculated from 100 simulated datasets using the participant-specific best-fitting parameters. Error bars: ±2 SD. For all observers, the Ideal-Confidence-Observer model had the highest confidence agreement, then the Basic-Probability model, then the remaining models. Red: confidence agreement of the observer. Green: predicted confidence agreement according to the best-fitting model for that observer. Black: predictions of the other models. Note that different observers had different maximum agreement counts depending on the number of repeated blocks (Figure 1B).

References

[1] Murphy KP. Conjugate Bayesian analysis of the Gaussian distribution. 2007;The University of British Columbia [Technical report]. [cited 2022 June 23]. Available from `https://www.cs.ubc.ca/~murphyk/Papers/bayesGauss.pdf`.

[2] Acerbi L, Ma WJ. Practical Bayesian optimization for model fitting with Bayesian Adaptive Direct

Search. Advances in Neural Information Processing Systems. 2017;30:1834–1844.

[3] Akaike H. A New Look at the Statistical Model Identification. IEEE Transactions on Automatic Control. 1974;19(6):716–723.

[4] Cavanaugh JE. Unifying the derivations for the Akaike and corrected Akaike information criteria. Statistics & Probability Letters. 1997;33(2):201–208.
